# Reconfigurable Ferroelectric‐Like Two‐Dimensional Electron Gas at Room Temperature

**DOI:** 10.1002/adma.74190

**Published:** 2026-07-30

**Authors:** Martando Rath, Yu Chen, Daniela Stornaiuolo, Aravind Raji, Alexandre Gloter, Antonio Cassinese, Andrea Rubano, Alessia Sambri, Domenico Paparo, Emiliano Di Gennaro, Jérôme Lecourt, Wilfrid Prellier, Francesco Rosa, Nicholas B. Brookes, Giacomo Ghiringhelli, Daniele Preziosi, Marco Salluzzo

**Affiliations:** ^1^ CNR‐SPIN Complesso Univ. Monte S. Angelo Naples Italy; ^2^ Department of Physics, University of Naples Federico II Complesso Univ. Monte S. Angelo Naples Italy; ^3^ Laboratoire de Physique de Solides, CNRS Université Paris‐Saclay Paris France; ^4^ Synchrotron SOLEIL, L'Orme des Merisiers Paris France; ^5^ CNR‐ISASI Complesso Univ. Monte S. Angelo Naples Italy; ^6^ Laboratoire CRISMAT, CNRS Université de Caen Normandie Caen France; ^7^ Dipartimento di Fisica Politecnico di Milano Milano Italy; ^8^ ESRF The European Synchrotron Grenoble France; ^9^ CNR‐SPIN Milano Italy; ^10^ CNRS, IPCMS Université de Strasbourg Strasbourg France

**Keywords:** ferroelectric material, ferroelectric switching, two‐dimensional electron gases

## Abstract

Engineering ferroelectric two‐dimensional electron gases (2DEGs) offers a promising route toward nonvolatile and reconfigurable electronic functionalities, yet room temperature implementations remain scarce. Here, we report a room temperature ferroelectric‐like 2DEG at the SrO‐${\mathrm{NbO}}_{2}$ interface between (001) ${\mathrm{SrTiO}}_{3}$ (STO) and $K_{0.5}{\mathrm{Na}}_{0.5}{\mathrm{NbO}}_{3}$ (KNN) thin films. Robust ferroelectricity is confirmed by piezoforce microscopy, optical second harmonic generation, Nb‐$M_{2,3}$/Ti‐$L_{2,3}$ edges x‐ray linear dichroism, scanning transmission electron microscopy, and macroscopic polarization measurements. The ferroelectric polarization enables nonvolatile control of interfacial transport and the realization of reconfigurable conducting channels with arbitrary geometry at room temperature. The coupling between ferroelectric order and 2DEG conductivity arises from the extension of the polarization into the interfacial unit cells hosting the electron gas. These results establish $K_{0.5}{\mathrm{Na}}_{0.5}{\mathrm{NbO}}_{3}$/${\mathrm{SrTiO}}_{3}$ heterostructures as a ferroelectric‐like oxide electronic platform, opening pathways for low‐power, nonvolatile electronic and spin‐orbitronic devices.

## Graphical abstract

We demonstrate the realization of a novel ferroelectric‐like two‐dimensional electron gas (2DEG) based on the epitaxial interface between SrO‐terminate SrTiO3 and ferroeletric ${\mathrm{KNb}}_{0.5}{\mathrm{Na}}_{0.5}O_{3}$ thin films. We show nonvolatile control of the transport properties enabling spatially reconfigurable devices in which conducting pathways are defined, removed, and rewritten via local ferroelectric polarization switching at room temperature.

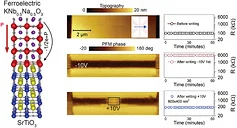

## 1 Introduction

During the past two decades, research on oxide two‐dimensional electron gas (2DEG) [1] has significantly raised expectations for all‐oxide electronics due to the wide range of emergent functional properties. These include unconventional superconductivity [2, 3, 4], gate‐tunable insulator‐to‐metal transitions [5], Rashba‐like spin–orbit coupling [6, 7], magnetism [3, 8, 9, 10], and high spin‐to‐charge conversion efficiency [11].

More recently, the discovery of oxide heterostructures that exhibit the coexistence of ferroelectric (FE) polarization with a 2DEG at low temperature enabled nonvolatile control of electronic properties [12, 13], such as conductivity [14, 15], spin transport [16], and even ferromagnetism [17], opening new pathways for innovations in low‐power electronics.

Despite this progress, FE‐2DEGs demonstrating robust nonvolatile behavior remain rare. Heterostructures that combine a band insulator (e.g., ${\mathrm{LaAlO}}_{3}$ (LAO) or ${\mathrm{AlO}}_{x}$) with Ca‐doped ${\mathrm{SrTiO}}_{3}$ (STO) exhibit a low FE‐Curie temperature (*T* 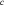 = 35 K) and weak polarization ( 2–5 μC/${\mathrm{cm}}^{2}$), restricting their use to cryogenic temperatures [14, 17]. Other 2DEGs based on FE layers embedded in the heterostructures, such as ${\mathrm{Ba}}_{0.5}{\mathrm{Sr}}_{0.5}{\mathrm{TiO}}_{3}$ [18], ${\mathrm{BiFeO}}_{3}$ [19, 20], and ${\mathrm{BaTiO}}_{3}$ [21, 22], have not yet demonstrated clear FE‐switching; instead, they typically behave as polar metals. Only recently, a FE‐2DEG characterized by a $T_{c}$ = 165 K has been realized by depositing ${\mathrm{AlO}}_{x}$ on high‐quality strained STO films [23].

Among the family of FE‐materials, $K_{0.5}{\mathrm{Na}}_{0.5}{\mathrm{NbO}}_{3}$ (KNN), due to its excellent room temperature piezoelectric and FE properties [24, 25, 26], is considered a promising lead‐free material for micro/nano‐electromechanical systems (M/NEMS), nonvolatile memory devices, and energy storage applications.

**FIGURE 1:**
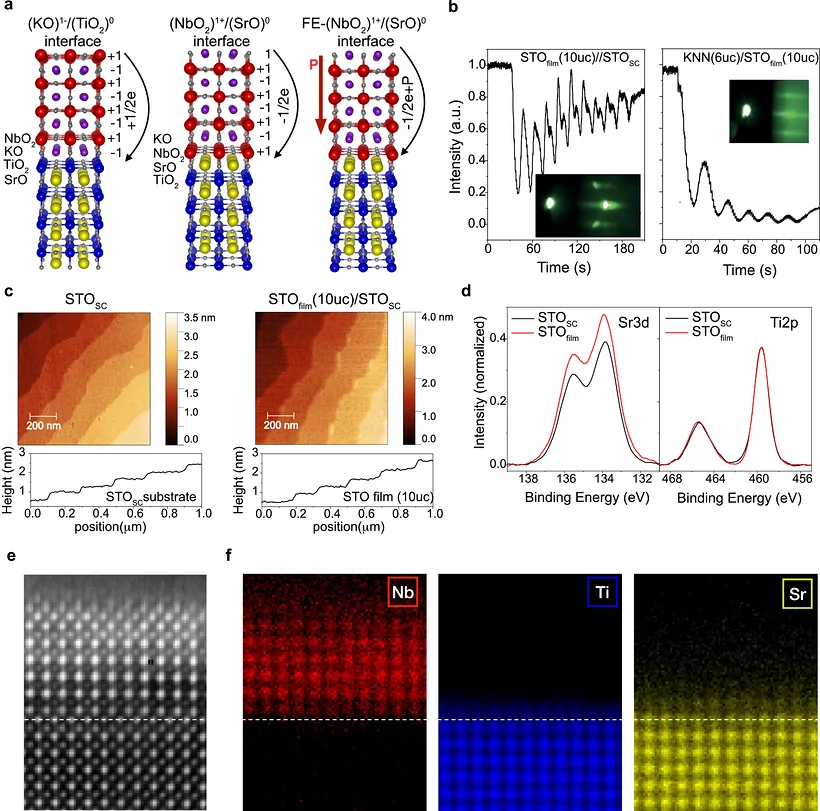
(a) Schematic of ideal electronic reconstruction mechanism to solve the polarization discontinuity by a charge‐transfer from the KNN to the STO interface in (001) STO/KNN heterostructures: (left panel) KO/${\mathrm{TiO}}_{2}$ interface; (middle panel) ${\mathrm{NbO}}_{2}$/SrO interface; (right panel) ${\mathrm{NbO}}_{2}$/SrO interface in presence of a KNN FE‐polarization, where the net polarization (*P* 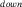 *P* <0, *P* 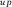 *P* >0) of the FE‐KNN changes the charge accumulated at the interface to a value equal to −0.5e+P. (b) RHEED specular spot oscillations during the growth of (left panel) an homo‐epitaxial ${\mathrm{STO}}_{film}$ (10 uc) on ${\mathrm{TiO}}_{2}$ terminated (001) ${\mathrm{STO}}_{SC}$, and (right panel) of a 6 uc KNN thin film on ${\mathrm{STO}}_{film}$(10 uc)/${\mathrm{STO}}_{SC}$. The insets show the RHEED patterns after the growth. (c) 1×1 $\mu m^{2}$ AFM topography and line profiles of BHF etched ${\mathrm{TiO}}_{2}$ terminated STO (001) single crystal (left panel) and of 10 uc ${\mathrm{STO}}_{film}$ (right panel), showing 1 uc high step terraces. (d) In situ Sr‐3*d* and Ti‐2*p* XPS core‐level spectra at take‐off angles of 40 degrees of the ${\mathrm{STO}}_{SC}$ substrate (black line) and of the 10 uc homoepitaxial STO thin film (red line). All spectra are normalized to the integral of the corresponding Ti‐2*p* core level spectra. (e) STEM‐HAADF image of the a‐LAO/KNN(6 uc)/${\mathrm{STO}}_{film}$/${\mathrm{STO}}_{SC}$ heterostructures and (f) Nb, Ti, and Sr EELS element maps across the interface. The data show that at the interface (sketched as dashed white line) the stacking is ${\mathrm{NbO}}_{2}$/SrO.

It features a high Curie temperature (*T* 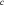 = 675 K) and a polarization at room temperature above 20 μC/${\mathrm{cm}}^{2}$ [25, 26]. First principles calculations predict the formation of a 2DEG at the (001) interface between ${\mathrm{KNbO}}_{3}$ and STO, which can be switched on and off by reversing the FE‐polarization of ${\mathrm{KNbO}}_{3}[$ 27]. As shown in Figure 1a, KNN consists of alternating (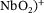 and (K(Na)O)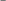 planes along the [001] direction. Within a polarization catastrophe scenario, a 2DEG is expected to form exclusively at the (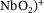/(SrO)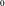 interface (Figure 1a, middle panel) [27], in contrast to the LAO/STO system, where a (LaO)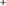/(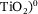 stacking is required to generate a 2DEG.

Moreover, theoretical studies predict a large polarization in the KNN layer, which can extend into the adjacent STO and naturally points toward the interface, enhancing the interfacial electron density (as shown in Figure 1a, right panel) [27]. Reversing the polarization direction is expected to substantially deplete this electron density. However, these predictions have yet to be experimentally verified, as epitaxial growth of KNN on SrO terminated STO, essential for this interface, is technically challenging.

Here, we demonstrate nonvolatile nanoscale control of a ferroelectric‐like two‐dimensional electron system, enabling spatially reconfigurable devices in which conducting pathways are written, erased, and rewritten via local ferroelectric polarization switching at room temperature.

The 2DEG is realized at the (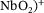/(SrO)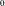 interface between (001) KNN films and SrO‐terminated STO grown by pulsed laser deposition (PLD). SrO termination is achieved by tuning the PLD deposition conditions for the growth of an homoepitaxial STO buffer layer (${\mathrm{STO}}_{film}$) with thickness ≥ 10 unit cells (uc) on a ${\mathrm{TiO}}_{2}$ terminated STO single crystal (${\mathrm{STO}}_{SC}$), in agreement with other studies [28, 29, 30].

In situ reflection high energy electron diffraction (RHEED), Ti‐2*p*/Sr‐3*d* core level x‐ray photoemission spectroscopy (XPS), and ex situ scanning transmission electron microscopy (STEM) with electron energy loss spectroscopy (EELS) confirm that the 2DEG forms at the SrO–${\mathrm{NbO}}_{2}$ interface.

Room‐temperature ferroelectric switching at the nanoscale is demonstrated by piezoresponse force microscopy and spectroscopy, while optical second‐harmonic generation (SHG), Nb–$M_{2,3}$ edge x‐ray linear dichroism (XLD), scanning transmission electron microscopy, and macroscopic polarization measurements provide converging evidence for the ferroelectric nature of the KNN layer within the heterostructures. Furthermore, STEM analysis and Ti–$L_{2,3}$ edge XLD reveal non‐centrosymmetric polar distortions in the interfacial STO unit cells. Nonvolatile switching of the 2DEG transport properties, together with back‐gating experiments, demonstrates that the interfacial electronic system is coupled to a switchable polar state, enabling nonvolatile control of interfacial transport beyond conventional ferroelectric field effect.

## 2 Results

The a‐LAO/KNN/${\mathrm{STO}}_{film}$/${\mathrm{STO}}_{SC}$ heterostructures were grown by RHEED‐assisted PLD in an oxygen partial pressure exceeding 10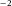 mbar (Experimental section). In Figure 1b, we show the intensity of the specular RHEED spot as a function of time during the sequential deposition of 10 uc homoepitaxial STO and of 6 uc KNN film. In both cases, the RHEED intensity exhibits distinct oscillations as a function of time, suggesting a layer‐by‐layer growth. The RHEED patterns after the growth (insets of Figure 1b) show a very good final crystalline quality of both STO and KNN films. After the KNN deposition, a uniform and flat a‐LAO protecting layer (5 uc), required to preserve the KNN film due to the high reactivity of alkali metals, is deposited at a lower temperature (Experimental section). The surface morphology measured by atomic force microscopy (AFM) shows atomically flat and uniform terraces, matching the step‐terrace morphology of the underlying STO single‐crystal substrate (see Figure S1).

In the following, we show that the emergence of a 2DEG in a‐LAO/KNN/${\mathrm{STO}}_{film}$/${\mathrm{STO}}_{SC}$ is correlated to the switching of termination in homoepitaxial ${\mathrm{STO}}_{film}$. The RHEED specular intensity versus time during the growth of 10 uc ${\mathrm{STO}}_{film}$ (Figure 1b, left panel) exhibits, after the deposition of the first STO layer, a double periodicity. The corresponding period is approximately half the time required for the deposition of a single unit cell. This indicates the emergence of SrO islands on the initially ${\mathrm{TiO}}_{2}$‐terminated substrate and gradual termination switching (see Figure S2 and Note S1). AFM measurements (Figure 1c) show that the 10 uc homoepitaxial ${\mathrm{STO}}_{film}$ is characterized by atomically flat terraces with unit‐cell‐high steps, confirming ordered and mostly single‐terminated surfaces.

The chemical surface termination is analyzed by comparing in situ Sr‐3*d* core‐level XPS spectra (Figure 1d, left panel) before (black line) and after (red line) the growth of the STO (10 uc) thin film, acquired at θ = 40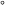 take‐off angle and normalized to the corresponding Ti‐2*p* integrated intensity. The increased intensity of the normalized Sr‐3*d* peak of the homoepitaxial ${\mathrm{STO}}_{film}$, compared to the bare substrate, shows a surface enrichment of Sr (see Figure S3 and Note S2). A main SrO termination is confirmed by the quantitative analysis of the Sr‐3*d* and Ti‐2*p* XPS intensities, following the methodology reported in ref. [30] (see Note S2).

To verify that the interface between the KNN and the STO film is ${\mathrm{NbO}}_{2}$/SrO in the full a‐LAO/KNN/ ${\mathrm{STO}}_{film}$/${\mathrm{STO}}_{SC}$ heterostructures, we performed high‐angle annular dark‐field (HAADF) and annular bright‐field (ABF) scanning transmission electron microscopy (STEM), complemented by electron energy loss spectroscopy (EELS). As shown in Figure 1e,f, the combination of HAADF‐STEM and EELS maps shows that the dominant interfacial stacking at the KNN/${\mathrm{STO}}_{film}$ interface is ${\mathrm{NbO}}_{2}$/SrO, as expected. Some traces of Nb/Ti intermixing are found in the interfacial unit cell of the ${\mathrm{STO}}_{film}$, while at the a‐LAO/KNN interface, we do observe some La diffusion into the top KNN layers, likely substituting lighter K/Na atoms (see Figure S4 and Note S3). The interface location and the ${\mathrm{NbO}}_{2}$/SrO stacking are confirmed by the analysis of combined HAADF and ABF STEM data, as shown in the Supporting Information (see Figure S5 and Note S3).

**FIGURE 2:**
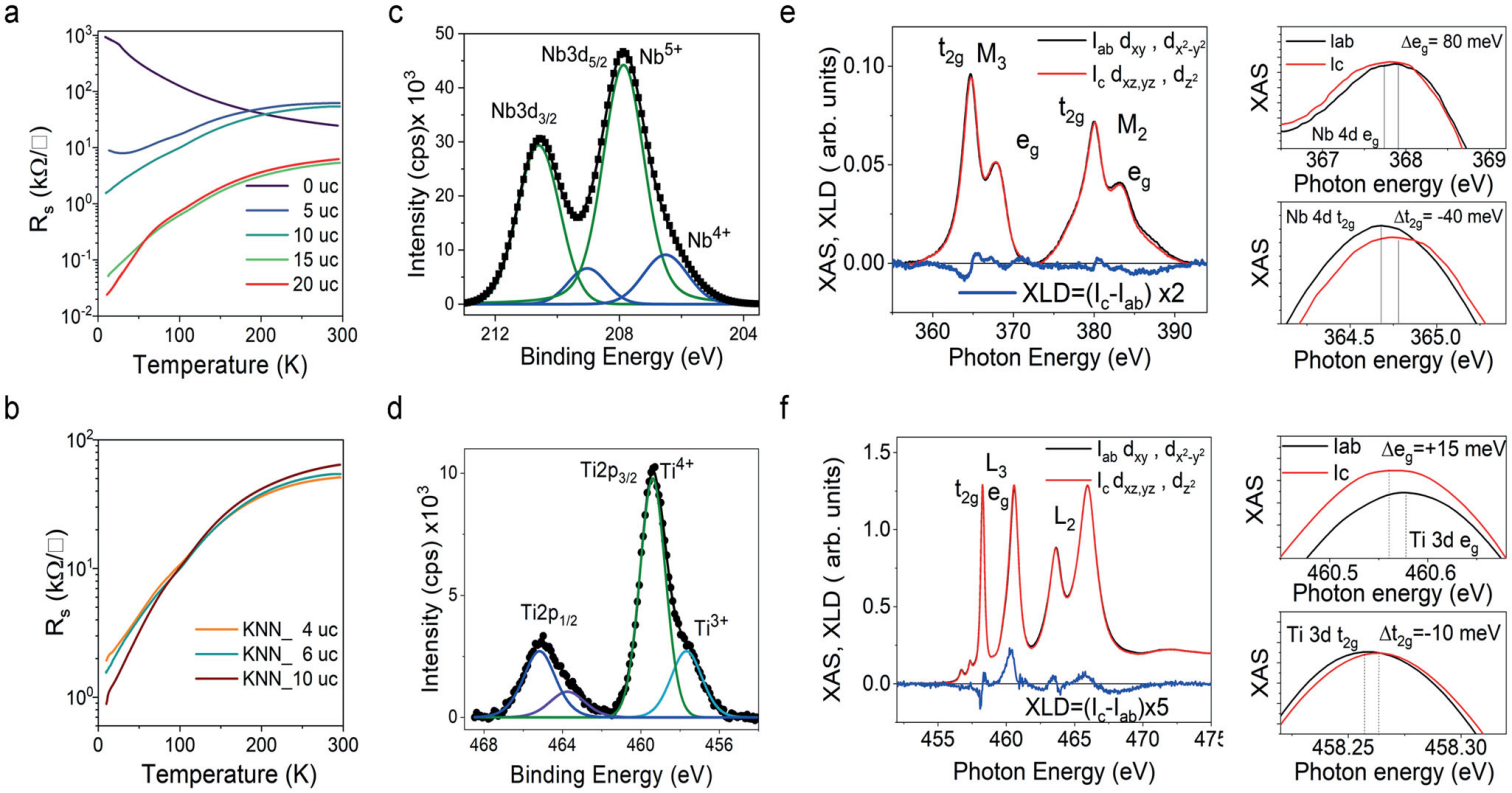
Temperature‐dependent sheet resistance of a‐LAO (5uc)/KNN/${\mathrm{STO}}_{film}$/${\mathrm{STO}}_{SC}$ heterostructures (a) as a function of the ${\mathrm{STO}}_{film}$ buffer layer thickness (0 uc no buffer layer) and (b) of the KNN thickness (with ${\mathrm{STO}}_{film}$ thickness of 10 uc). In situ (c) Nb‐3*d* and (d) Ti‐2*p* XPS spectra on the KNN(6uc)/${\mathrm{STO}}_{film}$/${\mathrm{STO}}_{SC}$, along with a fitting using multiple Voigt peaks, after standard Shirley background subtraction. The features at the lower binding energy in both Ti‐2*p* and Nb‐3*d* spectra indicate the presence of ${\mathrm{Ti}}^{3+}$ at the KNN/${\mathrm{STO}}_{film}$ interface and ${\mathrm{Nb}}^{4+}$ in the KNN film, respectively. Room temperature $I_{c}$ (red), $I_{ab}$ (black) XAS spectra and ($I_{c}$–$I_{ab}$) XLD (blue curve) spectra at the (e) Nb $M_{2,3}$ and (f) Ti $L_{2,3}$ edges of a‐LAO(5 uc)/KNN(6 uc)/${\mathrm{STO}}_{film}$ (10 uc)/${\mathrm{STO}}_{SC}$ 2DEG. The data show opposite energy shifts of the $M_{3}$ $e_{g}$ (e, upper right panel) and $t_{2g}$ (e, bottom right panel) peaks. Similarly, the Ti $L_{2,3}$ edge XAS spectra in (f) show the opposite shifts of $e_{g}$ (f, upper right panel) and $t_{2g}$ (f, bottom right panel) peaks acquired with the two linear polarizations. This result shows that both KNN and the interfacial STO unit cells are non‐centrosymmetric.

Figure 2a shows the temperature‐dependent sheet resistance of heterostructures as a function of the STO‐buffer layer thickness. Without a buffer layer, a‐LAO/KNN(6 uc)/${\mathrm{STO}}_{SC}$ samples exhibit a semiconducting behavior, whereas the a‐LAO/KNN(6 uc)/${\mathrm{STO}}_{film}$(10 uc)/${\mathrm{STO}}_{SC}$ trilayer is metallic down to 4 K. An intermediate STO buffer layer thickness (5 uc) yields a metallic state down to 40 K, below which the system becomes insulating. As shown in Figure 2b, varying the KNN thickness (*n* = 4, 6, 10) in a‐LAO/KNN(n)/${\mathrm{STO}}_{film}$(10 uc)/${\mathrm{STO}}_{SC}$ consistently yields metallic behavior down to low temperatures. The room temperature sheet resistance slightly increases with the KNN layer thickness, while at low temperature we do observe an opposite trend. To get insight into the electronic properties of the heterostructure, in situ core level Nb‐3*d* and Ti‐2*p* XPS was carried out on KNN/${\mathrm{STO}}_{film}$/${\mathrm{STO}}_{SC}$ without the a‐LAO protecting layer (Figure 2c,d). Nb‐3*d* and Ti‐2*p* spectra show main ${\mathrm{Nb}}^{5+}$ (3*d* 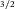 = 210.6 eV, 3*d* 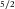 = 207.9 eV) and ${\mathrm{Ti}}^{4+}$ (2*p* 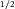 = 465 eV, 2*p* 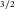 = 458.5 eV) doublets. However, peaks at lower binding energies in the KNN/${\mathrm{STO}}_{film}$/${\mathrm{STO}}_{SC}$ indicate the presence of ${\mathrm{Nb}}^{4+}$ and ${\mathrm{Ti}}^{3+}$ states. Given the short photoelectron escape depth (1‐3 nm), the observed ${\mathrm{Ti}}^{3+}$ signal originates from the interfacial STO layers, providing direct evidence for a 2DEG, similarly to other STO‐based oxide interfaces [31]. It is worth noting that XPS spectra on the whole heterostructure, including the 5 uc a‐LAO layer, show strongly reduced Ti 2*p* core level intensity, confirming the sensitivity to the interfacial ${\mathrm{STO}}_{film}$ layers and excluding contributions from the ${\mathrm{STO}}_{SC}$ in the measurement (see Figure S6 and Note S4). Additionally, occupied Nb‐4*d* states are also detected. XPS spectra acquired in shallow emission (sensitive to the topmost Nb cations), do not show any ${\mathrm{Nb}}^{4+}$ states, suggesting that these features are related to interfacial (inner) Nb cations (see Figure S7 and Note S4). The observation of both interfacial Ti‐3*d* and Nb‐4*d* states is consistent with ab initio calculations [27].

Figure 2e,f shows Nb‐$M_{2,3}$ and Ti‐$L_{2,3}$ edges x‐ray absorption spectroscopy (XAS) and XLD measurements, performed at the beamline ID32 of the European Synchrotron Radiation Facility (ESRF) [32]. Nb‐$M_{2,3}$ and Ti‐$L_{2,3}$ edges XAS probe unoccupied Nb‐4*d* and Ti‐3*d* states, respectively. The XLD spectra, that is, XAS spectra acquired with x‐ray linear polarization parallel ($I_{ab}$) or perpendicular ($I_{c}$) to the interface, can be used to determine the Nb‐4*d* and Ti‐3*d* orbital splitting and symmetry. Figure 2e shows the total electron yield (TEY) Nb‐$M_{2,3}$ linearly polarized XAS spectra of the KNN‐based 2DEG, acquired at room temperature. The spectra reveal distinct peaks at the $M_{3}$ and $M_{2}$ edges, corresponding to the 3*p* → 4*d* electronic transitions into the *t* 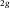 and *e* 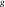 states. The key observation here is an anomalous orbital hierarchy: the *d* 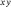 states ($I_{ab}$) lie at lower energy than the *d* 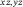 states ($I_{c}$), while the $d_{x^{2}-y^{2}}$ states ($I_{ab}$) are higher in energy than the $d_{z^{2}}$ states ($I_{c}$). In particular, by using atomic multiplet scattering calculations we find $\Delta t_{2g}$ = −45 meV and $\Delta e_{g}$ = +90 meV, where $\Delta t_{2g}$ and $\Delta e_{g}$ are defined as the energy difference between *d* 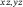 and *d* 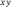 states, and between *d* 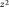 and *d* 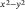 states, respectively (see Figure S8 and Note S5). This orbital splitting pattern is characteristic of non‐centrosymmetric distortions of the ${\mathrm{NbO}}_{6}$ octahedra and cannot be explained by simple tetragonal strain, which would produce $t_{2g}$ and $e_{g}$ splittings of the same sign. Similar anomalous XLD has been reported in prototypical ferroelectrics, such as ${\mathrm{BaTiO}}_{3}$ [33] and polar a‐LAO/${\mathrm{BaTiO}}_{3}$/${\mathrm{STO}}_{SC}$ 2DEG [22]. These results strongly indicate that the KNN layer is non‐centrosymmetric at room temperature, consistent with the presence of a ferroelectric order.

Interestingly, a qualitative comparable dichroic signature is observed at the Ti‐$L_{2,3}$ edge (Figure 2f), showing that the STO interfacial region, where the 2DEG is located, is characterized by non‐centrosymmetric polar‐distortions. In particular, the XLD spectra again show an inversion in orbital hierarchy: Ti‐3$d_{xy}$ states lie below the Ti‐3$d_{xz,yz}$ ones, and the Ti‐3$d_{z^{2}}$ states are lower in energy than the Ti‐3$d_{x^{2}-y^{2}}$ orbitals. By using atomic multiplet scattering calculations (see Figure S9 and Note S5), we find $\Delta t_{2g}$ = −15 meV and $\Delta e_{g}$ = +30 meV, smaller than the splitting observed for Nb *d*‐states indicating weaker polar distortions (Table S1), but just a factor 2–3 lower than the splitting observed at low temperature in polar ${\mathrm{BaTiO}}_{3}$ heterostructures [22]. Since TEY mode primarily probes Ti‐3*d* states in the layers at the interface with KNN, this result shows that the polar KNN film induces non‐centrosymmetric distortions in the STO unit cells where the 2DEG resides.

The XLD results are in excellent agreement with the structural analysis of polar distortions obtained from combined HAADF and ABF STEM imaging, which provides simultaneous contrast for heavy and light elements and enables a direct determination of the relative displacement of oxygen atoms with respect to neighboring cations (see Figure S5 and Note S3). This analysis reveals non‐centrosymmetric displacements consistent with a polarization oriented toward the interface in both the KNN layer and the interfacial STO unit cells.

In summary, combined transport, RHEED, AFM, XPS, and STEM‐EELS investigations demonstrate that a 2DEG forms at the (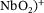/(SrO)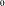 interface between KNN and buffer ${\mathrm{STO}}_{film}$ in a‐LAO/KNN/ ${\mathrm{STO}}_{film}$/${\mathrm{STO}}_{SC}$ heterostructures. The emergence of a metallic temperature dependence is strictly linked to the SrO termination of the STO buffer layer and is absent without such engineered termination. Moreover, XAS‐XLD data and STEM structural analysis show that KNN and the interfacial STO unit cells are non‐centrosymmetric and are characterized by FE‐dipoles pointing toward the interface in the KNN film and within the STO interfacial unit cells.

### 2.1 Ferroelectric Properties

In order to address the local FE‐properties of the a‐LAO/KNN/${\mathrm{STO}}_{film}$/${\mathrm{STO}}_{SC}$ heterostructures, we performed piezoresponse force microscopy (PFM) and switching spectroscopy PFM (SS‐PFM) measurements. With the sample interface grounded by Al bonding wires, FE‐domains were written by scanning the tip at a ±10 V DC bias voltage over concentric square patterns: an outer 5×5 $\mu m^{2}$ region with −10 V and an inner 3×3 $\mu m^{2}$ region poled with +10 V.

**FIGURE 3:**
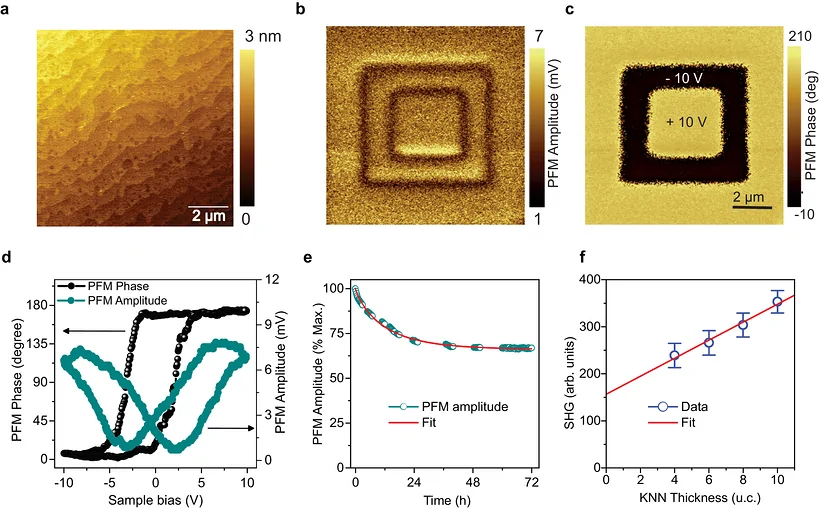
(a) Surface topography, (b) out‐of‐plane PFM amplitude and (c) PFM phase images of PLD grown a‐LAO (5 uc)/KNN (6 uc)/${\mathrm{STO}}_{film}$ (10 uc)/${\mathrm{STO}}_{SC}$ (001) film. The images are 8×8 $\mu m^{2}$. Square patterns were poled with −10 V DC bias applied to the 5×5 $\mu m^{2}$ region and +10 V DC bias applied to the 3×3 $\mu m^{2}$ region; the areas under the bright and dark squares illustrate the change of the polarization state after switching the as‐grown sample. (d) Local switching spectroscopy PFM phase (black scatter points, left scale) and PFM amplitude (dark cyan scatter points, right scale) versus bias voltage, showing clear nanoscale ferroelectric switching behavior. (e) Domain relaxation of the PFM amplitude as a function of time, along with a fit (described in the main text). (f) Optical second harmonic generation as a function of the KNN thickness. The blue points are the experimental data, and the error bars correspond to one standard deviation, while the red line is the corresponding linear fit.

Figure 3a–c shows the topography, out‐of‐plane PFM amplitude and phase images of a 8×8 $\mu m^{2}$ region, including the poled areas, for an a‐LAO/KNN(6 uc)/${\mathrm{STO}}_{film}$ (10 uc)/${\mathrm{STO}}_{SC}$ heterostructures. Dark areas in the phase image (0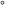) correspond to upward polarization (−10 V), and bright areas to downward polarization (+10 V). Notably, the unpoled regions, outside the inner 5× 5 $\mu m^{2}$ poled areas, show a 180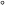 phase, indicating an intrinsic polarization pointing toward the interface, consistent with ab initio predictions [27] and with structural STEM analysis (see Figure S5 and Note S3). A clear 180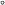 phase contrast between oppositely polarized domains is observed (Figure 3c), accompanied by a distinct amplitude response at the domain walls (Figure 3b), both characteristic features of ferroelectric materials. The results are confirmed in Figure 3d by local PFM hysteresis loops that show sharp 180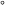 phase switching and symmetric butterfly‐shaped amplitude curves with negligible voltage offset, confirming robust, nanoscale ferroelectric switching. To rule out contributions from electrostatic charging, we monitored the time evolution of the PFM amplitude signal (Figure 3e) over 72 h. The signal slowly decayed, retaining 70% of its initial value, consistent with the relaxation dynamics of FE‐bound charges. This behavior follows a stretched exponential decay [34]: $\Delta P\left(t\right)=\Delta P_{0}\times e^{-\alpha t^{\beta }}$ where $\Delta P_{0}$ is the initial polarization, α is a decay constant, and β is the stretched exponent (0<β<1).

The slow relaxation of the PFM amplitude signal, together with the high‐oxygen pressure deposition conditions, strongly supports a true ferroelectric origin of the signal, as surface charge artifacts would decay much more rapidly to zero, and oxygen vacancy relaxation at room temperature occurs on much shorter time scales.

To further confirm the origin of the PFM results, we performed SHG measurements on a‐LAO/KNN(n)/ ${\mathrm{STO}}_{film}$(10 uc)/${\mathrm{STO}}_{SC}$ heterostructures, varying the KNN thickness (see Experimental sections and Note S6). SHG is widely applied to study the ferroelectric materials and FE‐transitions. A SHG signal is non‐vanishing in the dipole approximation when inversion symmetry is broken. This happens typically in the presence of an FE order parameter, but it can happen as well at the boundary between centro‐symmetric crystals, where the inversion symmetry is broken naturally by the presence of interfaces [35]. The data reveal, on the other hand, a clear linear increase of SHG intensity with the KNN thickness (Figure 3f). The linear fit intercept is nonzero, indicating a nonvanishing interfacial contribution coming from the 2DEG, while the dominant thickness‐dependent signal confirms that the SHG response increases linearly with the KNN layer, as expected in the case of ferroelectric KNN. It is worth noting that an alternative scenario, such as oxygen vacancies accumulation, does not fit with the room temperature sheet resistance and carrier density thickness dependence, which have an opposite trend.

Finally, we performed macroscopic ferroelectric measurements (see Figure S10 and Note S7) on capacitor structures fabricated by depositing 50 × 50 $\mu m^{2}$ Cr/Au top electrodes. Leakage‐compensated measurements reveal clear switching current peaks in the current versus voltage (*I*–*V*) characteristics and a well‐defined polarization hysteresis with a saturation polarization of 8 μC/${\mathrm{cm}}^{2}$.

Taken together, the combination of PFM hysteresis with long retention, SHG evidence of broken inversion symmetry, direct observation of polar ionic displacements at the atomic scale, and macroscopic polarization measurements provides a coherent, multi‐scale, and consistent demonstration of ferroelectricity of KNN.

### 2.2 Nanoscale Switching of the 2DEG Conductivity

**FIGURE 4:**
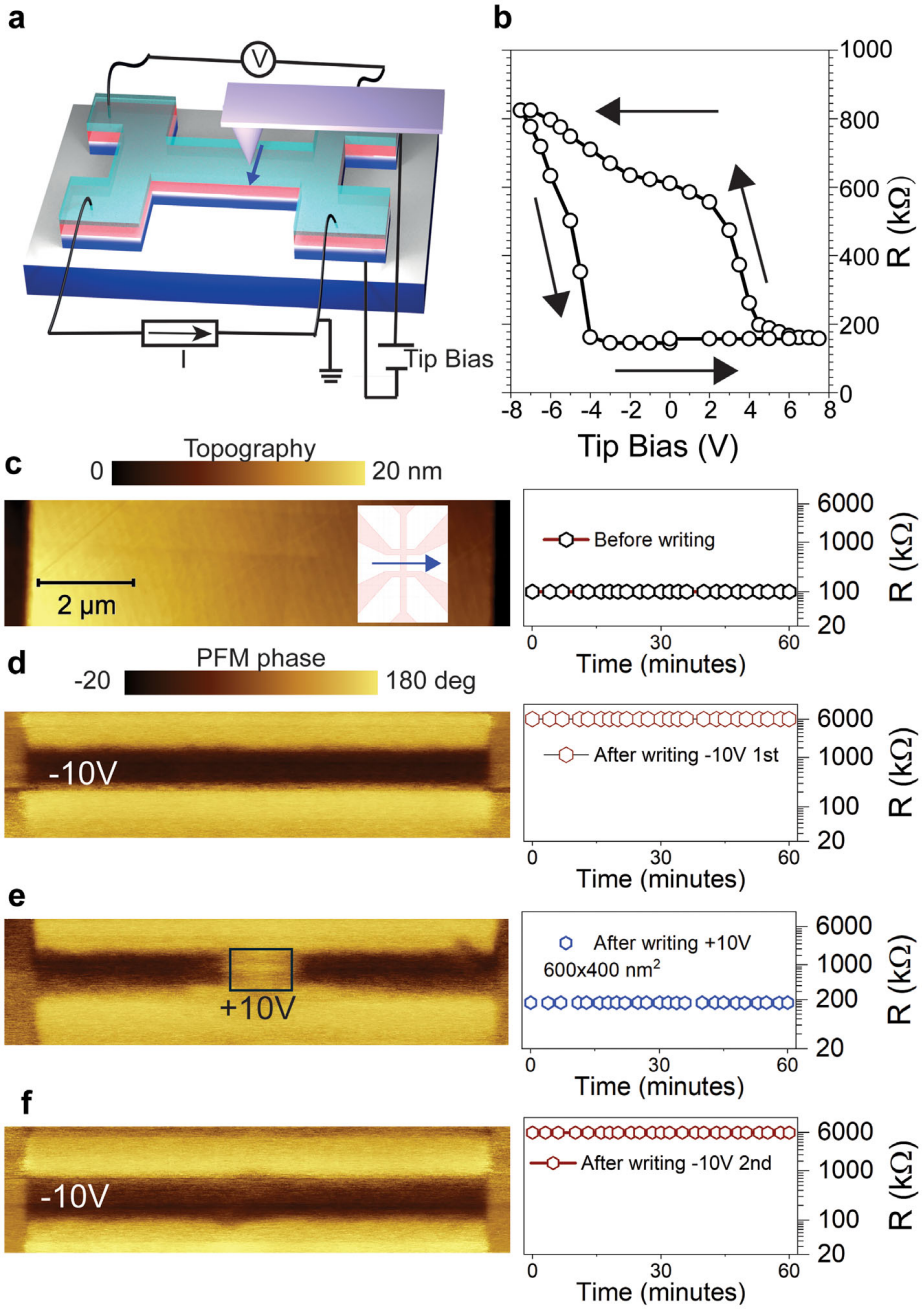
(a) Schematic of the device geometry, electrical configuration and corresponding (b) hysteretic dependence of the resistance of a 50 μm wide a‐LAO(5 uc)/KNN(6 uc)/${\mathrm{STO}}_{film}$(10 uc)/${\mathrm{STO}}_{SC}$ bridge as a function of tip bias during scanning along a single 45 μm line perpendicular to the channel (the arrow indicates the scan direction). The resistance of the bridge is measured by sourcing a current of 1 μA and measuring the voltage drop at the two other terminals in a pseudo‐four point probe geometry. (c) AFM topography of a 10 μm‐wide bridge (2 × 10 $\mu m^{2}$ scan area) and corresponding initial resistance (right), measured with a source current of 100 nA. Inset: schematic of the bridge geometry, with the arrow indicating the scan direction. (d) PFM phase image after writing a ∼10μm×400nm domain using −10 V tip bias ($P_{up}$), and corresponding time‐dependent resistance of the bridge. (e) PFM phase image after writing a central domain (∼600nm×400nm) using +10 V tip bias ($P_{down}$), and corresponding resistance versus time. (f) PFM phase image after re‐writing the ∼10μm×400nm domain using −10 V together with the corresponding resistance versus time.

To investigate the coupling between the ferroelectric KNN layer and the 2DEG at room temperature, we report in Figure 4 the results of local ferroelectric switching performed using a biased AFM tip on patterned a‐LAO(5 uc)/KNN(6 uc)/${\mathrm{STO}}_{film}$(10 uc)/${\mathrm{STO}}_{SC}$ (001) bridges with dimensions of 50 μm × 400 μm and 10 μm × 300 μm. Figure 4a shows a schematic of the 50 μm × 400 μm device, where the conducting AFM tip acts as a top electrode, enabling local control of the ferroelectric polarization.

In Figure 4b, we report the bridge resistance as a function of the voltage measured while scanning the biased AFM tip along a 45 μm line across the bridge, slightly smaller than its width. The data display a clear hysteretic behavior, associated with the gradual switching between *P* 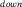 (positive tip bias) and *P* 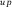 (negative tip bias) in the written region. The resistance changes by approximately a factor of five between the two polarization states.

To demonstrate almost complete interruption of the conduction path and the possibility to define and rewrite nanoscale conducting channels, we performed analogous experiments on narrower bridges (10 μm × 300 μm), as shown in Figure 4c–f. After writing a ∼10μm×400nm domain using a −10 V tip bias (Figure 4d, *P* 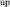), the bridge resistance increases from 100 kΩ to 6.5 MΩ, indicating a strong suppression of the 2DEG conductivity in the *P* 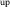 configuration.

In Figure 4e, a central *P* 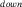 domain (∼600 nm × 400 nm) is subsequently written using a +10 V tip bias, resulting in the re‐establishment of a conducting path. A second writing cycle is shown in Figure 4f, where the extended *P* 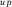 domain is redefined. Finally, the initial bridge resistance is reversibly restored after switching the ∼10μm×400nm domain to the *P* 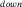 configuration.

This process can be repeated multiple times with reproducible results. It is worth noting that the sheet resistance of the nanoscale structure in Figure 4e is within 10% equal to the initial bridge sheet resistance as determined by calculating the corresponding geometrical shape factors using electromagnetic simulations (COMSOL). These experiments demonstrate nonvolatile and spatially controlled modulation of the oxide 2DEG, driven by the local ferroelectric polarization of the KNN layer, and enable the reversible definition and reconfiguration of nanoscale conducting pathways.

## 3 Discussion

**FIGURE 5:**
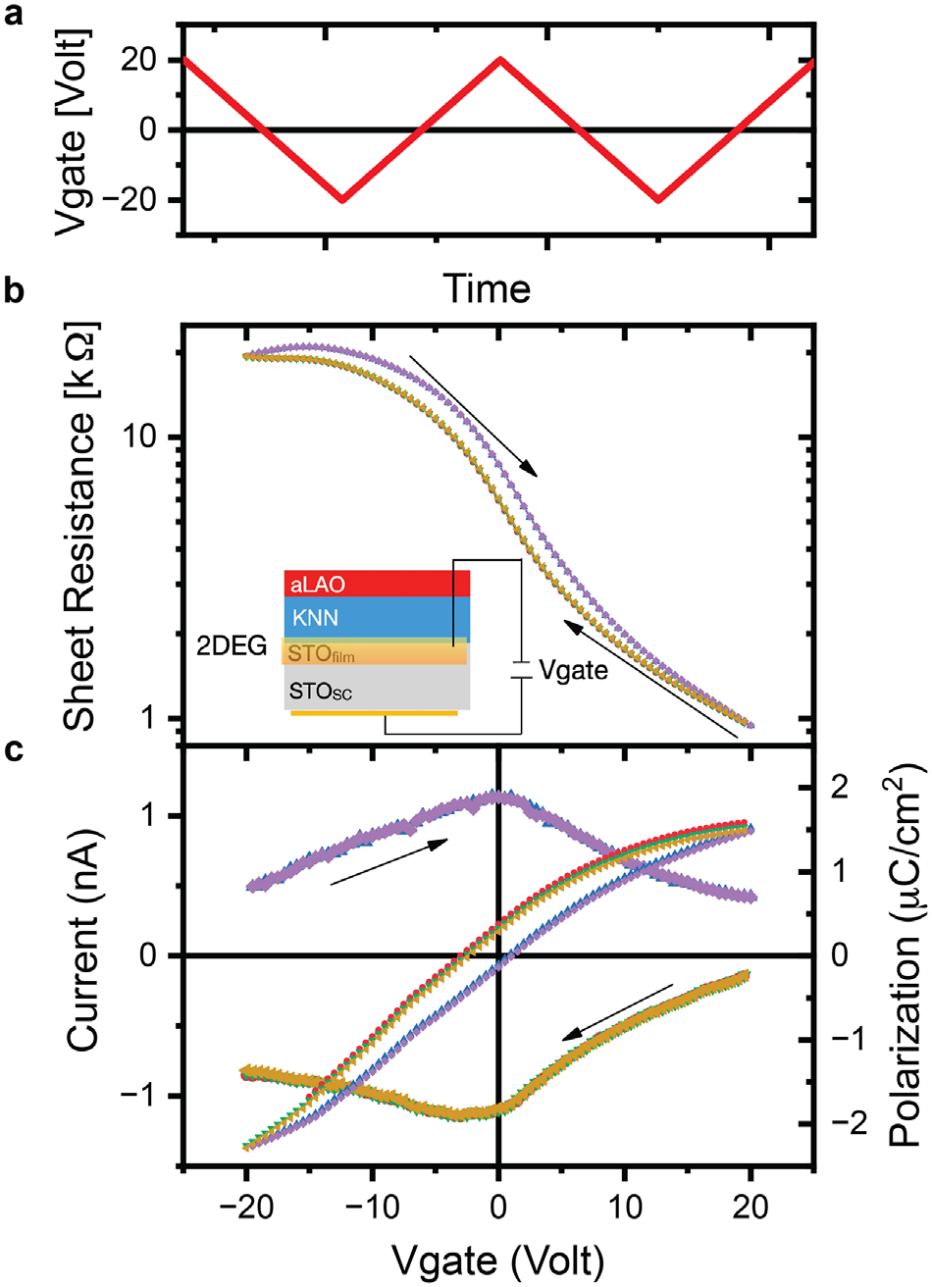
Back‐gate voltage experiment (a) Gate voltage versus time and corresponding gate voltage dependence of the (b) 2DEG sheet resistance (inset: contact configuration) and (c) Current across the capacitor structure. Arrows indicate the direction of the sweeping voltage. Clear switching current peaks are observed. In (c) right panel, we report the calculated polarization from the time‐integrated current.

In the present work, we report several experimental observations fully consistent with the theoretical prediction of ref. [27], which suggest the emergence of a ferroelectric 2DEG at the SrO/${\mathrm{NbO}}_{2}$ interface between STO and ${\mathrm{KNbO}}_{3}$, namely: the formation of interfacial ${\mathrm{Ti}}^{3+}$ and ${\mathrm{Nb}}^{4+}$ states; non centro‐symmetric (polar) local environment; the presence of finite polarization at room temperature in both KNN and STO interfacial unit cells; and the ferroelectric control of transport through polarization switching of KNN. However, a definitive proof of an intrinsic ferroelectric order parameter within the 2DEG itself requires the demonstration of switchable dipoles, within a region comprising the first interfacial KNN unit cell and the ${\mathrm{STO}}_{film}$, where the 2DEG is located. To have information about the possibility to switch the polarization within the 2DEG region, we performed a low‐temperature (5 K) back‐gating experiment in which the electric field is applied across the ${\mathrm{SrTiO}}_{3}$ substrate, while the interfacial 2DEG acts as the top electrode. In this configuration, the KNN FE‐layer is not involved in the switching process. Despite this, as shown in Figure 5, we observe a clear FE‐like hysteresis in the sheet resistance. Although hysteretic back‐gating effects are known in conventional STO‐based 2DEGs and are commonly attributed to carrier trapping and de‐trapping processes [36, 37], the response observed here exhibits important qualitative differences. In particular, after the initial “forming” process (i.e., the first positive polarization to +20 V), the sheet resistance is systematically lower during the backward sweep (from positive to negative gate voltage) than during the forward sweep (from negative to positive gate voltage), and the hysteresis remains highly reproducible over subsequent cycles. This behavior contrasts with that typically observed in non‐ferroelectric STO‐based 2DEGs, where hysteresis is generally associated with electron trapping and de‐trapping and often leads to higher resistance values during the backward sweep and to a progressive evolution of the hysteresis upon repeated cycling.

Moreover, the current flowing across the capacitor exhibits well‐defined switching peaks, which are absent in conventional trapping‐driven phenomena and instead indicate the presence of a switchable polar state. The existence of this polar state is further confirmed by the polarization‐versus‐gate‐voltage curves obtained both by integrating the displacement current (Figure 5c) and by independent ferroelectric analyzer measurements (see Figure S11). Taken together with the direct observation of polar distortions by STEM and XLD, these results indicate that the transport hysteresis is closely linked to the switching of an interfacial polar state coupled to the 2DEG, while carrier trapping effects may contribute only as a secondary mechanism, particularly during the initial forming process.

The persistence of an FE‐polarization and of a sheet resistance hysteresis in the absence of direct poling of the KNN layer demonstrates that a switchable polar state exists in a region, which includes the STO and/or the interfacial (STO/KNN) unit cells where the 2DEG is located. Importantly, the switching occurs at low voltages (< 20 V, less than 0.4 kV/cm) across a 0.5 mm thick ${\mathrm{STO}}_{SC}$. The ${\mathrm{STO}}_{SC}$ is quantum paraelectric at 5 K, not ferroelectric, while electric field‐induced ferroelectricity in STO requires switching gate voltages far beyond 20 V [16]. This excludes the ${\mathrm{STO}}_{SC}$ as the region where a polarization switching takes place.

On the other hand, the electric field is expected to be significantly enhanced in the interfacial ${\mathrm{STO}}_{film}$ due to its reduced thickness and lower effective dielectric constant. Combined with the STEM evidence of dipoles and the XLD signatures of non‐centrosymmetric breaking of inversion symmetry, this strongly indicates that the switching occurs within the interfacial region where the 2DEG resides.

These results suggest that the interfacial electron system is not merely modulated as in a ferroelectric field effect system, but is intrinsically coupled to and participates in a switchable polar state. In this sense, the 2DEG itself is part of a ferroelectric system.

The convergence of spectroscopic, structural, and transport evidence strongly supports the realization of a ferroelectric‐like 2DEG at the KNN/STO interface.

## 4 Conclusion

The realization of a room‐temperature FE‐like 2DEG at the $K_{0.5}{\mathrm{Na}}_{0.5}{\mathrm{NbO}}_{3}$/${\mathrm{SrTiO}}_{3}$ (STO) interface marks a significant advance toward the development of functional oxide‐based applications. A key finding of this work is the confirmation of the importance of the polarization catastrophe instability in the 2DEG formation at (001)‐oriented ${\mathrm{ABO}}_{3}$/STO oxide interfaces, even when one of the layers is a bulk ferroelectric. The difference in formal valence of the A‐site cations (K, Na in KNN; La in LAO) and the B‐site cations (Nb in KNN; Al in LAO) drives electron accumulation at the ${\mathrm{BO}}_{2}$/SrO interface in the KNN/STO heterostructure and at the more conventional AO/${\mathrm{TiO}}_{2}$ interface in the LAO/STO system. Notably, this electronic reconstruction occurs despite common interface nonidealities such as cation intermixing and potential oxygen vacancies, highlighting the critical role of polarization discontinuity in stabilizing the 2DEG. The data also show that the electron charge is distributed across the interface in both interfacial KNN and STO layers, as suggested by the core‐level XPS data demonstrating the presence of occupied Ti‐3$d^{1}$ and Nb‐4$d^{1}$ states, in agreement with ab initio calculations. While the transport properties of the FE‐like 2DEG formed at the KNN/STO interface show similarities with other STO‐based systems, the possibility that Nb‐4*d* electrons participate in the conduction is particularly intriguing. Indeed, Nb‐4*d* states are characterized by significantly stronger atomic spin–orbit coupling (SOC) than Ti‐3*d* orbitals with potential implications for spin‐orbitronic devices.

From a fundamental standpoint, the coexistence of switchable ferroelectric polarization and itinerant carriers in two dimensions remains a topic of ongoing debate. Our structural, transport and spectroscopic data show clear evidence that the ferroelectric KNN film induces polar distortions and electric dipoles in the adjacent STO layer where the 2DEG resides. Owing to the direct coupling between the ferroelectric order in KNN and the interfacial electron gas, this system offers a unique opportunity to locally switch the 2DEG on and off in a nonvolatile manner, even at room temperature (as shown in Figure 4). This capability enables a viable route toward the realization of reconfigurable nanoscale devices using AFM bias switching of KNN ferroelectric domains.

Altogether, these results open up the favorable prospect of exploiting spin/orbital transport phenomena and nonvolatile spin/orbit to charge conversion effects in low power electronic technologies, establishing the KNN/STO FE‐2DEG as a versatile platform for the development of novel, reconfigurable spin‐orbitronic and electronic devices.

## 5 Experimental Sections

### 5.1 Sample Growth

Epitaxial $K_{0.5}{\mathrm{Na}}_{0.5}{\mathrm{NbO}}_{3}$ (KNN)/${\mathrm{SrTiO}}_{3}$(STO)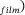/STOsc heterostructures were grown by RHEED‐assisted pulsed laser deposition (PLD) on ${\mathrm{TiO}}_{2}$ terminated STO substrates. A STO single crystal and a polycrystalline KNN (prepared by standard solid‐state reaction) were used as targets. To compensate for the volatility of K and Na during calcination and sintering, 5 mol % excess $K_{2}{\mathrm{CO}}_{3}$ and ${\mathrm{Na}}_{2}{\mathrm{CO}}_{3}$ were added during KNN target synthesis. The details of the KNN ceramic target synthesis and its functional properties in the thicker film are described in Refs. [38, 39]. The KNN layer thickness was varied from 4 to 10 unit‐cells (uc), while the STO buffer layer thickness ranged from 0 (no buffer) to 20 uc. The homoepitaxial ${\mathrm{STO}}_{film}$ was deposited at 750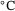 under an $O_{2}$ partial pressure of 1$\times 10^{-2}$ mbar, while optimized KNN films were grown at 600 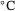 under 5$\times 10^{-2}$ mbar $O_{2}$. Both targets were ablated using a laser frequency of 1 Hz and a fluence of 1.3 J/${\mathrm{cm}}^{-2}$ with substrate to target distance of 40 mm and elliptical laser spot size of 1 mm × 0.5 mm. Under these conditions, the formation of oxygen vacancies in either layers is unfavorable. A 5 uc amorphous a‐LAO capping layer was deposited at 500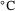 in 5$\times 10^{-5}$ mbar $O_{2}$, and then the samples were cooled down to room temperature in 5$\times 10^{-2}$ mbar of background oxygen pressure.

### 5.2 Device Fabrication

LAO(5 uc)/KNN(6 uc)/${\mathrm{STO}}_{film}$(10 uc)/${\mathrm{STO}}_{SC}$ bridges with dimensions of 50 μm × 400 μm and 10 μm × 300 μm were fabricated using a combination of optical lithography and cold ion milling. The purpose of these bridge structures was to investigate room temperature coupling between ferroelectric polarization and electronic transport, with particular attention to the emergence of nonvolatile, polarization‐control of the conductivity at the interface. The detailed fabrication procedure, including lithographic patterning, etching parameters, and device processing steps, is provided in Ref. [40].

### 5.3 X‐Ray Photoelectron Spectroscopy

To identify more directly the atomic termination, in situ core‐level x‐ray photoelectron spectroscopy (XPS) measurements were employed. All spectra were recorded using Al Kα excitation (hν = 1486.6 eV) source in ultra‐high‐vacuum ($<10^{-10}$ mbar) using a SCIENTA OMICRON operated in a large area mode, using hemispherical energy analyzer, adjusted at a pass energy of 50 eV. The XPS spectra were background corrected with Shirley functions and fit using CASA‐XPS software.

### 5.4 Piezoresponse Force Microscopy

PFM and SS‐PFM measurements were carried out in contact mode using a Park Systems XE‐100 AFM, equipped with Cr/Au‐coated NC36 tips (Nanosensors). The sample interface was grounded via Al bonding wires, and FE domains were written by scanning the tip with ± 10 V DC bias over concentric square patterns: an outer 5 × 5 $\mu m^{2}$ region poled with −10 V, and an inner 3 ×3 $\mu m^{2}$ region with +10 V. An external lock‐in amplifier (SRS Stanford Research Systems) applied an AC bias (*f* = 11 kHz) to measure both the phase and amplitude of the local piezoelectric response.

### 5.5 Optical Second Harmonic Generation

Optical second harmonic generation (SHG) measurements were performed at room temperature using a COHERENT Ti: Sapphire pulsed laser system. For SHG measurements, we employed light pulses having a central wavelength of 800 nm, a pulse width of about 40 fs, and a repetition rate of 1 kHz. The laser beam was directed through a variable neutral density filter and a linear polarizer set to p‐polarization, then focused onto the sample surface using a 20 cm focal‐length lens. After interaction with the sample, the beam was passed through a short‐pass filter to remove the fundamental 800 nm wavelength, re‐collimated with a lens, and directed into a monochromator and photomultiplier. The detected signal was integrated over frequency and polarization to maximize the signal‐to‐noise ratio. The pulse energy was varied but always kept below 50 μJ per pulse on a 80 μm spot size to prevent sample damage. The SHG signal was verified to scale quadratically with the pump power and to exhibit a narrow spectral line, ensuring the absence of spurious contributions. To eliminate systematic effects, the samples were mounted multiple times in random order, confirming that the observed signal trends were independent of spot size on the sample surface.

### 5.6 High Resolution Transmission Electron Microscopy

Cross‐sectional transmission electron microscopy (TEM) lamellae were prepared using a focused ion beam (FIB) technique at C2N, in the University of Paris‐Saclay, France. Around 20 nm of amorphous carbon was deposited on top for protection prior to the FIB lamellae preparation. The HAADF, ABF, and EELS imaging were done in a NION UltraSTEM 200 C3/C5‐corrected scanning transmission electron microscope (STEM). The experiments were done at 200 keV with a probe current of approximately 12 pA and convergence semi‐angles of 30 mrad. The EELS data were acquired with a MerlinEM detector (Quantum Detectors Ltd) in a 4×configuration (1024 × 256) installed on a Gatan ENFINA spectrometer mounted on the microscope [41].

### 5.7 X‐Ray Absorption Spectroscopy and X‐Ray Linear Dichroism

Nb $M_{2,3}$ and Ti $L_{2,3}$ edges x‐ray absorption spectroscopy (XAS) and x‐ray linear dichroism (XLD) measurements were performed at the beamline ID32 of the European Synchrotron Radiation Facility (ESRF). During the experiments, the sample was mounted in a vertical orientation and maintained under ultrahigh‐vacuum conditions. The XLD data were acquired in the total electron yield (TEY) mode using linearly polarized x‐rays incident at an angle θ = 60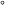 with respect to the surface normal. TEY Nb $M_{2,3}$ and Ti $L_{2,3}$ edges spectra were obtained as the difference between the average of 8 XAS spectra, acquired with x‐ray linear polarization perpendicular ($I_{c}$) and parallel ($I_{ab}$) to the interface, respectively.

### 5.8 Macroscopic Polarization Measurements

Polarization–electric field measurements were performed using a TF Analyzer (AixACCT 2000) on capacitor structures fabricated by depositing 50 × 50 $\mu m^{2}$ Cr/Au top electrodes. In order to reduce/eliminate the influence of leakage current in low thickness dielectric/ferroelectric capacitor structure, we have used the dynamic leakage current compensation (DLCC) module of the analyzer. The DLCC mode is based on two ac measurements at adjacent frequencies to subtract leakage, and it is routinely used in ultrathin or leaky ferroelectric materials.

## Conflicts of Interest

The authors declare no conflicts of interest.

## Supporting information


**Supporting File**: adma74190‐sup‐0001‐SuppMat.pdf.

## Data Availability

Data needed to evaluate the conclusions of this paper are present in the Letter and its Supporting Information. Additional data are available from the corresponding authors upon reasonable request.
