# Supplementary material for: Reconfigurable Ferroelectric‐Like Two‐Dimensional Electron Gas at Room Temperature
Source: Adv Mater. 2026 Jul 30;38(52):e74190. doi: 10.1002/adma.74190 (PMC13579201; doi:10.1002/adma.74190)
Supplement: Supplementary file 1 [file ADMA-38-e74190-s001.pdf]

Supplementary information

Reconfigurable ferroelectric-like two-dimensional electron gas at room temperature

Martando Rath[1*], Yu Chen[1], Daniela Stornaiuolo[2,1], Aravind Raji[3,4], Alexandre Gloter[3], Antonio Cassinese[2,1], Andrea Rubano[2,5], Alessia Sambri[1], Domenico Paparo[2,5], Emiliano Di Gennaro[2,1], Jérôme Lecourt[6], Wilfrid Prellier[6], Francesco Rosa[7], Nicholas B. Brookes[8], Giacomo Ghiringhelli[7,10], Daniele Preziosi[9], and Marco Salluzzo[1*]

[1*]CNR-SPIN, Complesso Univ. Monte S. Angelo, Naples, I-80126, Italy.
[2]Department of Physics, University of Naples Federico II, Complesso Univ. Monte S. Angelo, Naples, I-80126, Italy.
[3]Laboratoire de Physique de Solides, CNRS, Université Paris-Saclay, Orsay, Paris, F-91190, France.
[4]Synchrotron SOLEIL, L'Orme des Merisiers, Gif sur Yvette, Paris, F-91405, France.
[5]CNR-ISASI, Complesso Univ. Monte S. Angelo, Naples, I-80126, Italy.
[6]Laboratoire CRISMAT, Université de Caen Normandie, CNRS, UMR 6508, 6 Boulevard Maréchal Juin, Caen, F-14000, France.
[7]Dipartimento di Fisica, Politecnico di Milano, Piazza Leonardo da Vinci 32, Milano, I-20133, Italy.
[8]ESRF, The European Synchrotron, 71 Avenue des Martyrs, Grenoble, F-38043, France.
[9]Université de Strasbourg, CNRS, IPCMS, UMR 7504, Strasbourg, F-67034, France.
[10]CNR-SPIN, Piazza Leonardo da Vinci 32, Milano, I-20133, Italy.

*Corresponding author(s). E-mail(s): martando.rath@spin.cnr.it ; marco.salluzzo@spin.cnr.it;

Table of Contents

Note S1. Supplementary RHEED data.

Note S2. Analysis of Sr-3*d* XPS core level and quantitative estimation of the surface termination

Note S3. Supplementary EELS, HAADF and ABF STEM data.

Note S4. Location of Ti^{3+} and Nb^{4+} states from XPS analysis.

Note S5. Atomic multiplet scattering calculations of Nb $M_{2,3}$-edge and Ti $L_{2,3}$-edge XAS and XLD.

Note S6. Description of Optical Second Harmonic Generation (SHG).

Note S7. Macroscopic ferroelectric measurements.

Figure S1. AFM topography.

Figure S2. RHEED analysis of Homoepitaxial STO_{film}.

Figure S3. Analysis of *in-situ* XPS Sr 3*d* core-level.

Figure S4. Combined EELS and HAADF-STEM analysis of KNN/STO interface.

Figure S5. ABF+HAADF data and analysis

Figure S6. Analysis of Ti 2*p* XPS core level peaks.

Figure S7. Analysis of Nb 3*d* XPS core level peaks.

Figure S8. XAS, XLD atomic multiplet scattering simulation of Nb $M_{2,3}$-edge spectra

Figure S9. XAS, XLD atomic multiplet scattering simulation of Ti $L_{2,3}$-edge spectra

Figure S10. Macroscopic ferroelectric characterization of KNN films

Figure S11: Macroscopic ferroelectric characterization in back gate configuration

Table S1. Crystal field parameters used for the XAS and XLD simulations shown in the Figures S6 and S7.

Table S2: KNN thickness dependence of the transport properties at room temperature

Supplementary References

Figure S1. **AFM topography.**

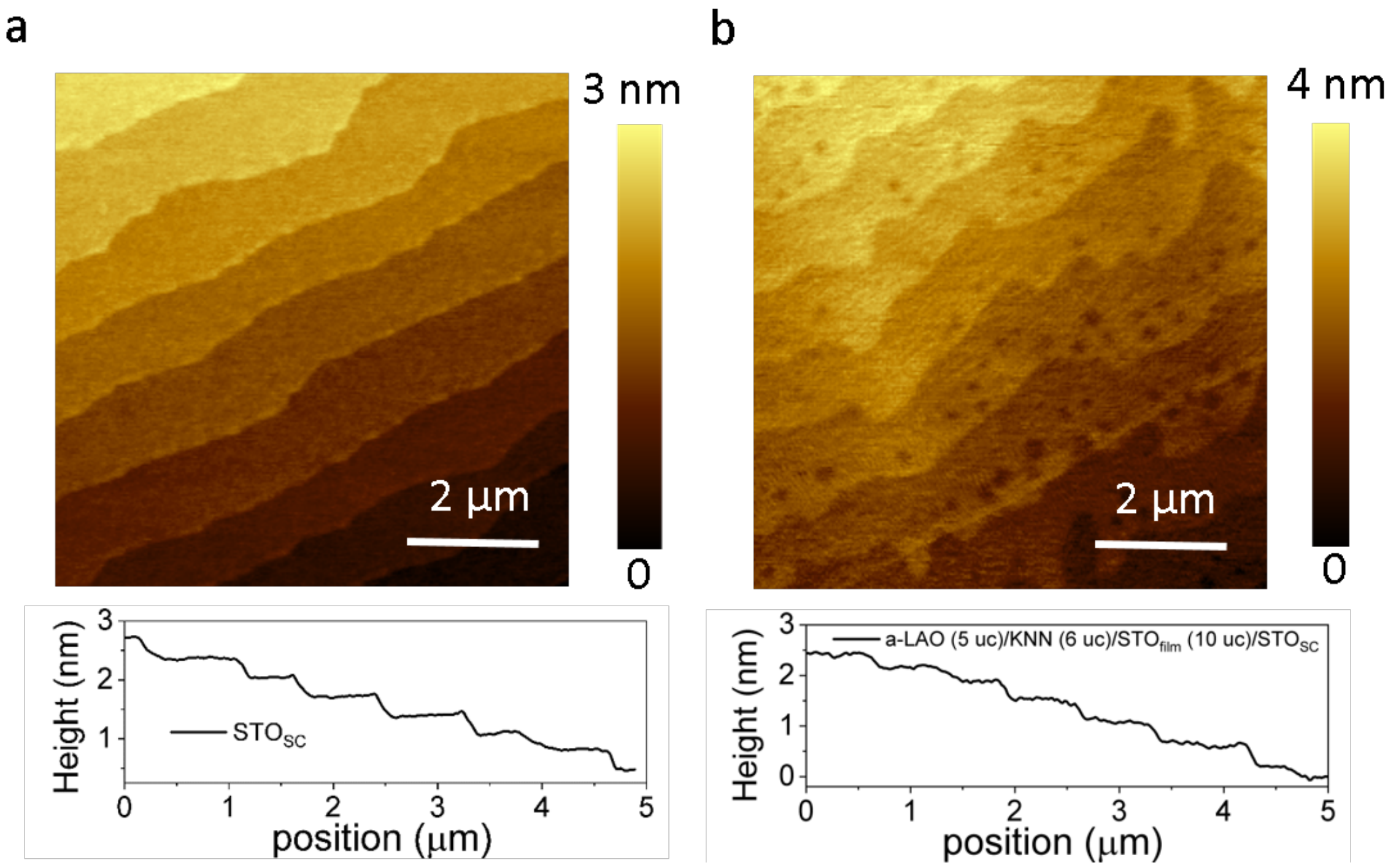

Figure S1: AFM topography. Non-contact AFM images on a 5x 5 μm^2 area of (a) BHF treated TiO_2 terminated STO substrate and (b) an a-LAO (5 uc)/KNN (6 uc)/STO_{film} (10 uc)/STO_{SC} heterostructures. The data show an atomically flat step-terrace morphology in both cases, with a unit-cell step height even after the deposition of the a-LAO amorphous layer.

Note S1: Supplementary RHEED data

The intensity of the specular (0 0) and first-order (0 1) diffraction RHEED spots during the deposition increases as a function of time (see Figure S2a) and exhibit a periodic modulation reflecting the ordering and disordering processes associated with layer-by-layer growth. The persistence of oscillations over multiple periods indicates a coherent growth.

The intensity ratio between the specular and diffracted spot at the end of the growth (see Figure S2b) decreases. This result is a further indication of a termination switching from TiO_2 to SrO, since it is well known that the (0 1) structure factor has a minimum for TiO_2 termination and a maximum for SrO termination (Ti is much lighter than Sr).

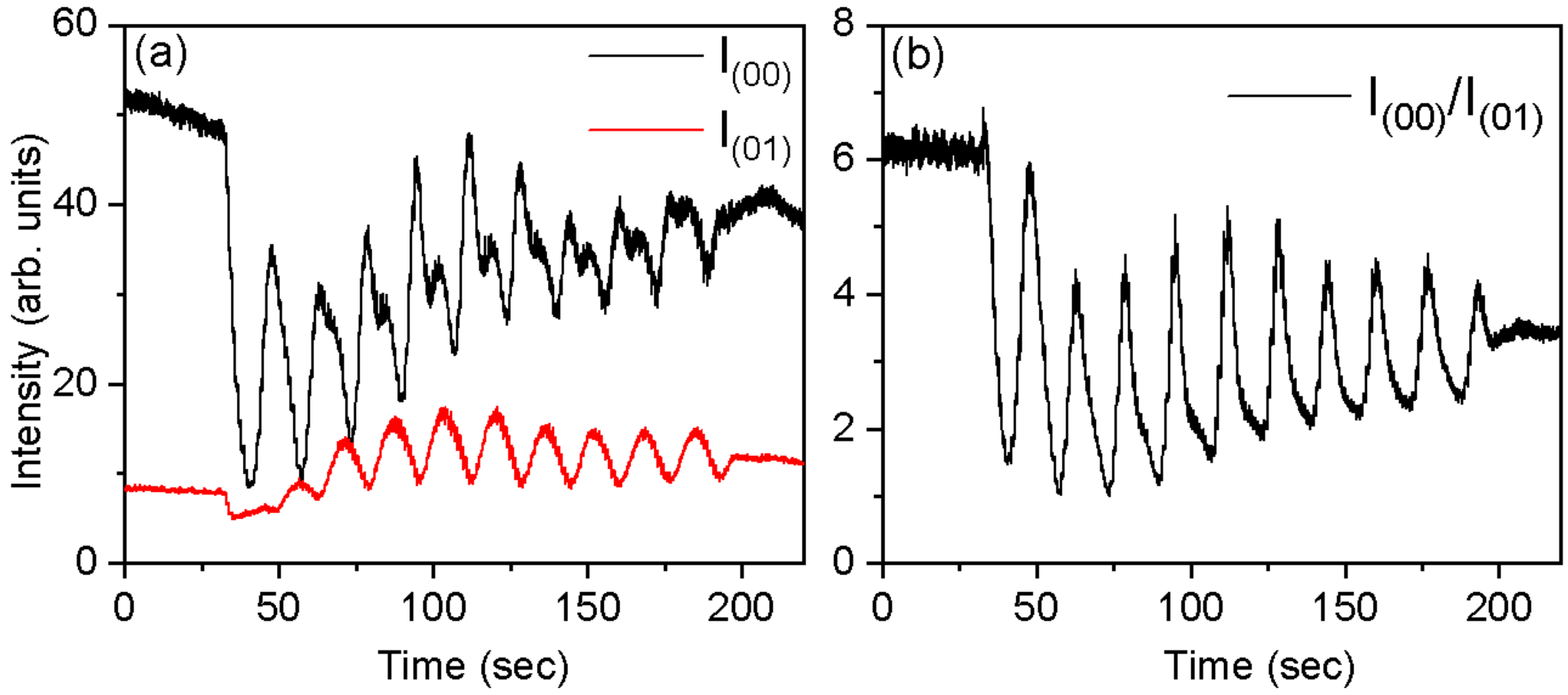

Figure S2: RHEED analysis of Homoepitaxial STO_{film}. (a) RHEED Intensity for (00) specular spot ($I_{(00)}$) and for first order (01) diffraction spot ($I_{(01)}$), and (b) Ratio between the intensity of the (00) specular RHEED spot and first order (01) diffraction spot as a function of time during the growth of 10 unit cells thick $SrTiO_3$ (STO) buffer layer on TiO_2 terminated STO (001) single crystal substrate.

Note S2: Analysis of Sr-3*d* XPS core level and quantitative estimation of the surface termination

Further indication of a SrO termination of the 10 unit cells STO_{film} grown on a TiO_2-terminated STO substrate comes from the comparison of *in-situ* Sr 3*d* core-level XPS spectra and relative Sr 3*d* and Ti 2*p* intensities of a BHF-etched TiO_2-terminated STO substrate and of the homoepitaxial STO film (10 u.c.) grown on a TiO_2-terminated STO (001) single-crystal substrate. In this study, all XPS spectra were background-corrected using a Shirley background and fitted with pseudo-Voigt line shapes using CASA-XPS software.

Analysis of the Sr *3d* core-level line shape:

Sr 3*d* core-level spectrum consists of two main spin–orbit split components: The Sr $3d_{5/2}$ peak at approximately 133.7 eV and the Sr $3d_{3/2}$ peak at approximately 135.6 eV. For the BHF-etched TiO_2-terminated STO substrate, the Sr 3*d* spectrum exhibits two sharp peaks, consistent with a single chemical environment of Sr and characteristic of bulk Sr contributions. In contrast, the Sr 3*d* spectrum of the homoepitaxial STO_{film} (10 u.c.) grown on the TiO_2-terminated STO substrate (see Figure S3b) show, in addition to the two main bulk related peaks, two additional, broader components at higher binding energies, shifted by approximately 1.1 eV. The presence of this components is attributed in literature (see for example supplementary

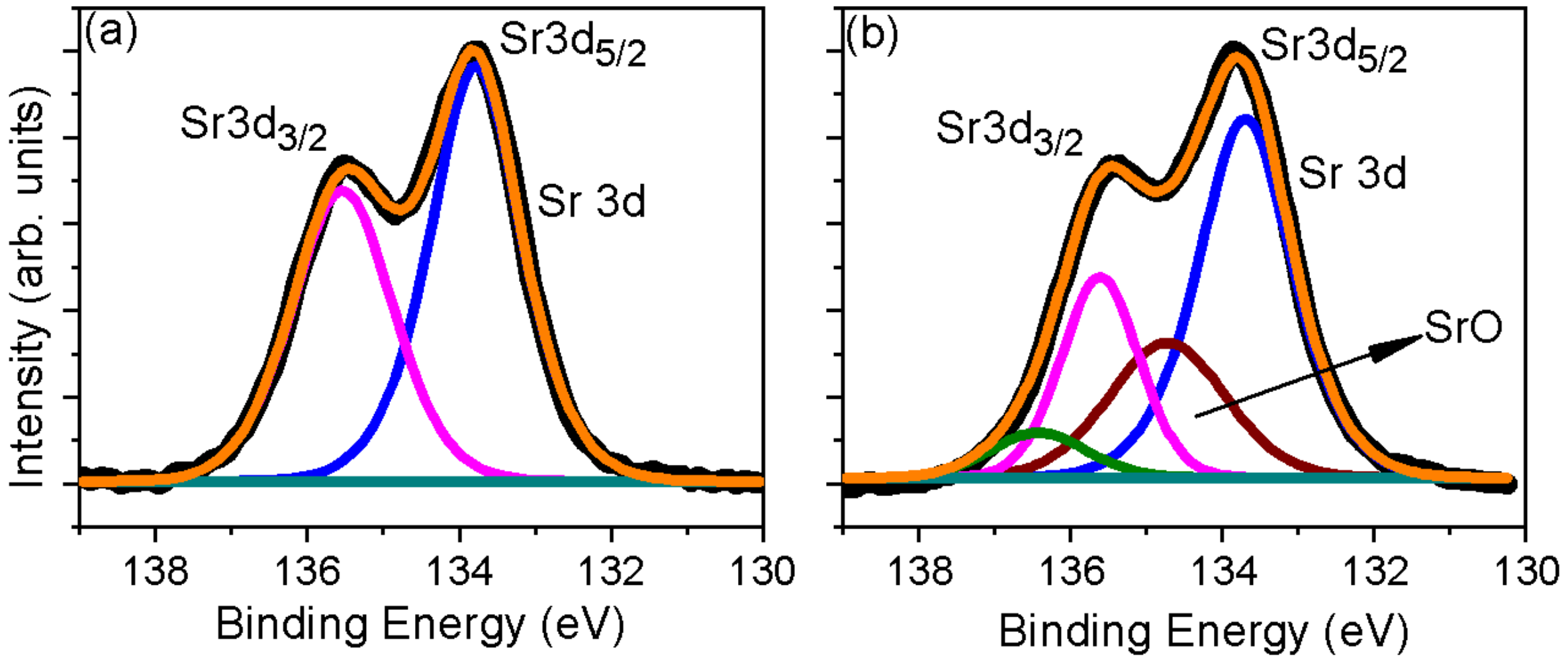

Figure S3: Analysis of *in-situ* XPS Sr 3*d* core-level. (a) The deconvolutions into Sr-bulk more intense doublets (Sr $3d_{3/2}$ and Sr $3d_{5/2}$) for BHF etched TiO_2terminated STO (001) single crystal and (b) The Sr-surface (binding energy position for SrO peak shows at higher binding energies) contributions lower intensity doublets in addition to Sr-bulk for homo-epitaxial STO_{film} (10 u.c.) on TiO_2 terminated STO.

Refs. [1, 2]) to the emergence of Sr at the surface which is either bond to other ions at the surfaces (in the latter case in particular when the surfaces are exposed to air) or are differently coordinated respect the bulk, which apply in our case (here Sr as one missing coordinated oxygen at the surface respect the bulk).

Estimation of the SrO surface coverage:

To assess more quantitatively the SrO coverage of our STO_{film}, we can rely on the expression derived in ref. [3], which allow to establish the SrO coverage from the intensities of Sr-3*d* and Ti-2*p* peaks:

$$\mathrm{I}_{Sr}/(\mathrm{I}_{Ti}+\mathrm{I}_{Sr}) = \frac{\left(A + B * e^{\left(-\frac{a}{2\lambda_{Sr}\mathrm{Cos}(\vartheta)}\right)}\right) N_{Sr}\,\sigma_{Sr}}{\left(A + B * e^{\left(-\frac{a}{2\lambda_{Sr}\mathrm{Cos}(\vartheta)}\right)}\right) N_{Sr}\,\sigma_{Sr} + \left(B + A * e^{\left(-\frac{a}{2\lambda_{Ti}\mathrm{Cos}[(\vartheta)}\right)}\right) N_{Ti}\,\sigma_{Ti}}$$

where A is the SrO termination coverage, B= 1-A is the TiO_2 termination coverage, ϑ the angle between the surface normal and the analyzer, λ_{Sr} and λ_{Ti} are Sr and Ti escape depths for the considered core levels, N_{Sr} and N_{Ti} are the average number of Sr and Ti ions within the inner layers (we assume $N_{Sr}=N_{Ti}$ here) and σ_{Sr}, σ_{Ti} are the sensitivity factors which depend on the calibrated transmission functions and its energy dependence of our spectrometer.

The theoretical expected values for full SrO and TiO_2 terminations are $I_{Sr}/(I_{Sr} + I_{Ti}) = 0.55$ and $I_{Sr}/(I_{Sr} + I_{Ti}) = 0.49$. From our data we obtain $I_{Sr}/(I_{Sr} + I_{Ti}) = 0.56 \pm 0.01$ for the STO_{film} , confirming main SrO termination, and $I_{Sr}/(I_{Sr} + I_{Ti}) = 0.50 \pm 0.01$ for the STO single crystal, confirming main TiO_2 termination.

Note S3: Supplementary EELS, HAADF and ABF STEM data

Interface Stacking:

Figure S4a displays combined EELS chemical map of LAO/KNN/STO_{film}/STO_{SC} heterostructures. the data show the dominant NbO_2/SrO interfacial stacking at the KNN/STO_{film} interface, the presence of some Nb/Ti intermixing at the KNN/STO_{film} interface and of La diffusion into the KNN layers at the a-LAO/KNN interface. Figure S4b-c shows HAADF-STEM image of KNN/STO_{film} interface with the area (solid line rectangle) from which EELS image was carried out. This HAADF-STEM + EELS data show that the heterostructures, including an STO_{film} buffer layer the interface is mostly NbO_2/SrO, as expected.

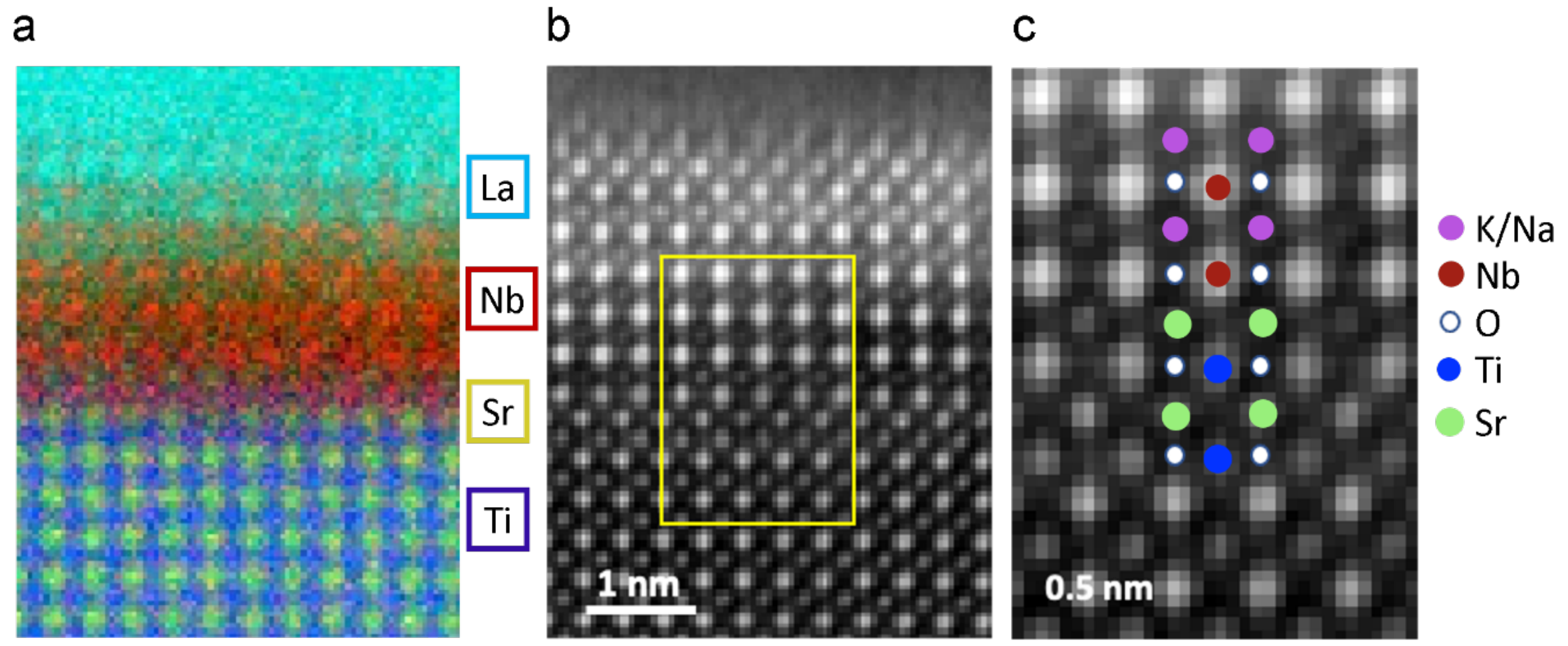

Figure S4: Combined EELS and HAADF-STEM analysis of KNN/STO interface. (a) Combined EELS element map across the interface and (b) corresponding STEM-HAADF image of the KNN/STO interface region of a-LAO/KNN/STO_{film}//STO_{SC} heterostructures. (c) A magnified HAADF image from the highlighted region in (b), with an atomic model obtained from the element map, indicating the SrO/NbO_2 interface.

To further assess the interface stacking and to study polar distortions, we combined simultaneous HAADF (High-resolution high-angle annular dark field) and ABF (Annular Bright-Field) Scanning Transmission Electron Microscopy (STEM) images to get contrast for both heavy and light elements. ABF-STEM images, even if more prone to noise and artifacts

due to aberration effects and a weaker signal-to-noise ratio, nicely complements the partial view obtained from cation-only observations using HAADF. This allows to get information about the atomic stacking complementary to the EELS chemical map shown in the manuscript. Moreover, it also allows to get information about polarization within both KNN and STO layers, from the relative displacements of cations and oxygen ions.

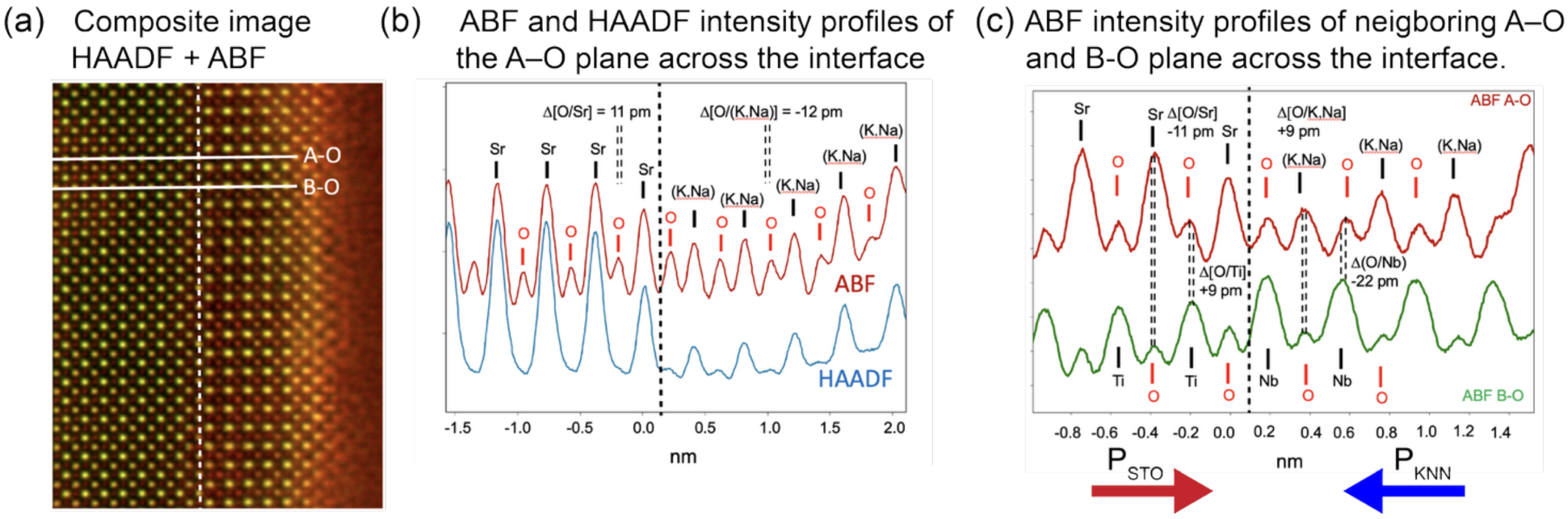

Figure S5: ABF+HAADF data and analysis: (a) Composite HAADF+ABS image. The HAADF image (in green) is overlaid with the inverted ABF image (in red). In this representation, cation atoms appear yellow-green, while the oxygen atoms, visible in ABF, appear in red. (b) ABF (red) and HAADF intensity profiles of the A-O planes. (c) ABF intensity profiles of neighbouring A-O (red) and B-O (green) planes. In (a)-(c) the vertical dashed line indicates the interface location.

In Fig. S5a we show a composite image in which the HAADF image (in green) is overlaid with the inverted ABF image (in red). This representation causes the cation atoms to appear yellow-green, depending on their relative intensity in HAADF and ABF, while the oxygen atoms, visible only in ABF, appear in red. Intensity profiles, shown in Fig. S5b,c, were extracted for the A-O and B-O planes across the interface (white lines in (a)). To improve the signal-to-noise ratio, these profiles were obtained by averaging over five adjacent planes. In Figure S5b, the red curve corresponds to the ABF average profile and reveals both the A-site cations (either Sr or (K,Na)) and the oxygen atoms. The HAADF profile (shown in blue) clearly highlights only the A-site cations. In Figure S5c, ABF profiles are compared for A–O and B–O planes, obtained from two neighbouring planes. From these data we can clearly identify the interface from the location of Sr-O and (K,Na)O planes. It turns out that they are separated by a NbO_2 plane, which agrees well with EELS map and with a SrO/NbO_2 interface stacking.

Polar distortions from HAADF+ABF data:

From the combined ABF and HAADF data we can also study the polar distortions in both KNN and STO interfacial unit cells. Despite the lower positional accuracy of ABF for atomic columns, we fitted the maxima of both A- and B- site cations and oxygen atoms to determine the relative displacement of the oxygen columns with respect to the neighbouring A-A and B-B cations. A key result is that in the A–O planes, the oxygen atoms position when compared to the average of the position of the neighbouring Sr-Sr pairs are displaced backward in the last $SrTiO_3$ unit cell (noted as a Δ[O/Sr] with a value of approximately 11 pm). It is indicating that the polarization vector points toward the interface, in agreement with the HAADF analysis. In the KNN region, the oxygen atoms are clearly displaced toward the right side of the Nb-Nb pairs, indicating ferroelectric polarization pointing toward the interface.

The analysis is confirmed in Fig.S5c, where ABF profiles are compared for A–O and B–O planes, obtained from two neighbouring planes. Although the signal-to-noise ratio is lower, this allows a direct comparison of the cation and oxygen positions within the whole unit cell. For instance, it shows that near the interface, the oxygen atoms in the Sr–O planes are located slightly backward compared to the nearby Ti atoms. This also indicates that the polarization points toward the interface for the last $SrTiO_3$ unit cell. In a similar manner, the displacement of the Nb atoms with respect to the nearby oxygen in the B–O planes are indicating a polarization toward the interface in the KNN side.

To summarize, this analysis confirms that the interface is located at the Sr–NbO_2- (K, Na) unit cell. Besides, both cation and oxygen displacements (in both A and B planes) indicate well-developed KNN polar distortion directed toward the interface. The last $SrTiO_3$ unit cells also shows substantial polar distortions toward the interface and a polarization also pointing towards the interface.

Note S4: Location of Ti^{3+} and Nb^{4+} states from XPS analysis

An analysis of the contribution from different layers to the Ti-2*p* core level spectra follows the standard attenuation formalism. The intensity of photoemitted electrons is given by:

$$I(\theta) = A\, G(\theta, \phi) \int_0^{+\infty} R(z)\; e^{-z/\lambda \cos\theta}\, dz$$

where λ is the inelastic mean free path (IMFP) of the emitted electrons. For Ti 2*p* photoelectrons, $\lambda \approx 1$ nm, as reported in [4], a value consistent with National Institute of Standards and Technology (NIST) database. However, values of the order of $\lambda \approx 2$ nm are also reported.

The factor A contains the attenuation from the top layers, in our case KNN, which is largely energy-independent in the relevant binding energy range for Ti 2*p* core-levels, and therefore does not affect the relative spectral weight of Ti^{3+} and Ti^{4+} components.

Assuming a simplified rectangular depth profile, one can separate the contributions from the STO film and the underlying STO substrate. Under our experimental conditions this analysis yields a ratio:

$$\frac{I_{\mathrm{film}}}{I_{\mathrm{substrate}}} \approx 8\text{–}11$$

indicating that the dominant contribution to the Ti 2*p* signal arises from the STO film (≥70–90%).

A further experimental confirmation comes from a comparison between the data acquired on the heterostructure before and after the a-LAO layer (5 uc) deposition, as shown below (Fig. S6). The Ti 2*p* core-level signal is a factor 10 lower and barely visible, with only 5 uc of a-LAO added. This demonstrate the sensitivity of the technique to only upper STO (STO_{film} layers) and exclude that the Ti^{3+} signal measured is due to the STO_{SC}, but it is due to states that are formed within the interfacial layers.

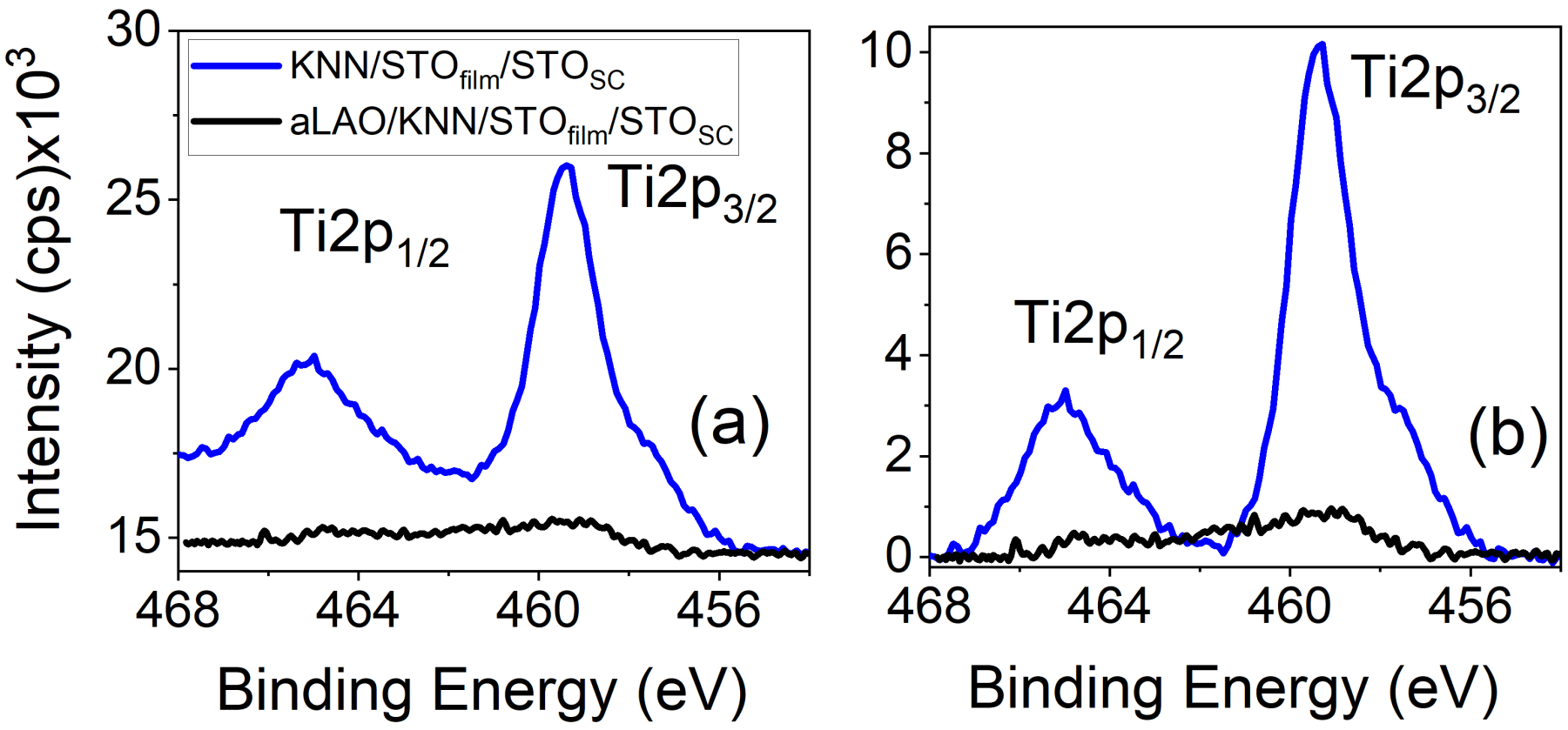

Figure S6: **Analysis of Ti 2*p* XPS core level peaks**: Comparison between KNN (6 uc)/STO_{film} (10 uc)/ /STO_{SC} (blue line) and a-LAO (5 uc)/KNN (6 uc)//STO_{film} (10 uc)//STO_{SC} Ti 2*p* XPS core level spectra (a) before and (b) after Shirley background subtraction.

Concerning Nb^{4+} states, we compare XPS spectra acquired at normal (more sensitive to inner Nb cations) and shallow emission angles (sensitive to the surface and topmost Nb cations). The data are shown in Fig. S7, and show that the Nb^{4+} contribution disappears in the shallow emission conditions. This analysis shows that, in addition to the Ti^{3+} component associated with the $SrTiO_3$ side, the Nb^{4+} signal is enhanced in more bulk-sensitive conditions, indicating that it originates from Nb atoms located in the interfacial KNN layers.

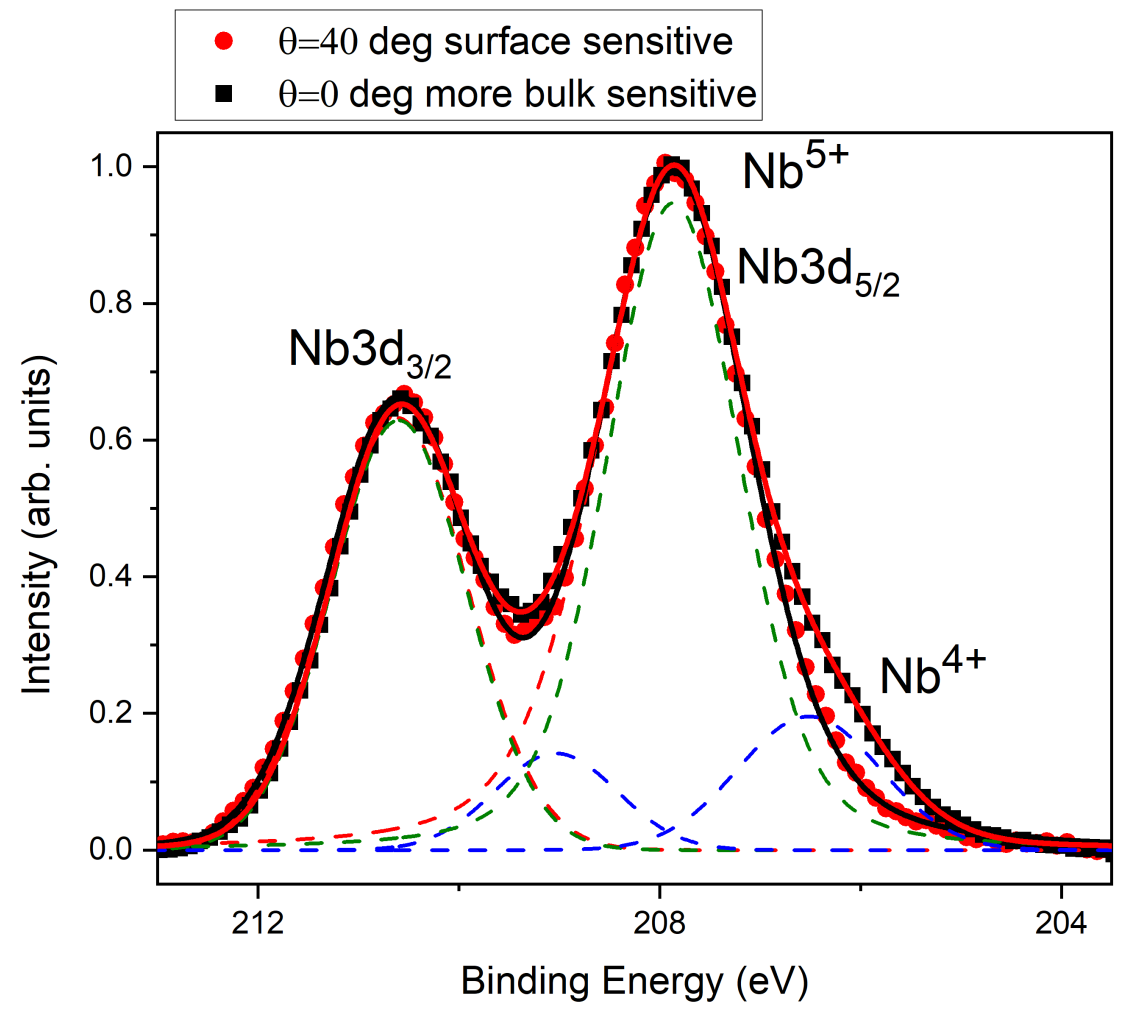

Figure S7: Analysis of Nb 3*d* XPS core level peaks: Comparison between Nb 3*d* core level XPS spectra in normal emission (θ=0 deg, black squares) and in the more surface sensitive shallow emission (θ=40 deg, red circles) condition. Continuous lines are fittings using multple Voigt peaks functions (red for θ=0 deg data, black θ=40 deg data). Dashed lines are the decomposition of the fitting curves in each component. For the θ=40 deg data only one pair of peaks corresponding to Nb^{5+} features (red lines) are needed to fit the data, while two pairs, one for Nb^{5+}(green lines) and one for Nb^{4+} features (blue lines), are necessary.

Note S5: Atomic multiplet scattering calculations of Nb $M_{2,3}$-edge and Ti $L_{2,3}$-edge XAS and XLD

Atomic multiplet scattering simulations were performed using ligand field atomic multiplet theory, implemented in the CTM4XAS software [5]. Atomic multiplet simulations at the Nb-$M_{2,3}$ edge and Ti-$L_{2,3}$ edge shown in Figures S8 and S9 were performed using Nb^{5+} and Ti^{4+} oxidation states in D4h symmetry, with Slater Integrals reduced by 90% respect the corrected Hartree-Fock values. In the case of Ti-XAS simulation, a charge transfer (CT) term was taken into account for the hybridization between Ti-3*d* and O-2*p* states in the TiO_6 cluster, *i.e.* a $3d^0$+$3d^1\underline{L}$ configuration, where $\underline{L}$ indicates a hole in the O-2*p* band. The CT is included in an Anderson impurity type of model where two parameters are necessary to describe the system: the energy difference between the Ti-*3d* and the ligand band (called Δ here), and the difference U_{pd}-$\underline{U}_{dd}$ between the core hole potential (U_{pd}) and the Hubbard *U* value (U_{dd}) for Ti-*d* electrons. Here we used, Δ=5 eV and U_{pd}-$\underline{U}_{dd}$ = 2 eV. Finally, the transfer integrals used were 2 and 1 for b_1, a_1 (d_{x2-y2}, d_{z2}) and b_2, e (d_{xy}, $d_{xz,\ yz}$) orbitals, respectively. This choice is commonly used for octahedral or close to octahedral systems, and the reasoning behind is that the probability for electron transfer is higher (in this case two times higher) for orbitals pointing in the bond direction, as d_{x2-y2}, d_{z2}.

Details of the simulation parameters are listed in Table S1.

The broadenings parameters used to reproduce the spectra were 0.1 eV for the Gaussian broadening, to account for the energy resolution, and different Lorentzian broadening (half width at half maximum) for each of the 4 peaks of the spectra, namely (from the first to the last): 0.7 eV, 1.4 eV, 0.8 eV, and 1.4 eV in the case of Nb $M_{2,3}$ edge XAS spectra, and 0.1 eV, 0.26 eV, 0.45 eV, and 0.42 eV in the case of Ti-XAS spectra peaks.

Ion	$10D_q$ (eV)	D_s (meV)	D_t (meV)	Δt_{2g} (meV)	Δe_g (meV)
Nb^{5+} fit Non-centrosymmetric distortion	3.4	6.43	12.86	-45	+90
Nb^{5+} tetragonal centrosymmetric distortion	3.4	-19.3	-25.7	-45	-90
Ti^{4+} fit Non-centrosymmetric distortion	2.0	2.14	4.29	-15	+30
Ti^{4+} tetragonal centrosymmetric distortion	2.0	-8.60	-6.43	-15	-30

Table S1: **Crystal field parameters from the Fit of data in the Figures S8 and S9**; Δt_{2g} is the difference between the energy of $d_{xz,yz}$ and d_{xy} states, while Δe_g is the difference between d_{z2} and d_{x2-y2} states.

In the Figures S8a and S8b, we show the average Nb-$M_{2,3}$ edge XAS and XLD data (black line), normalized to the M_3-edge XAS intensity, and corresponding calculated spectra reproducing the data (red line). In Figure S8b we also show the calculated XLD expected in the case of simple tetragonal distortion (green line), which will give rise to a Δt_{2g} and Δe_g splitting of the same sign (see table S1).

Similarly, in Figures S9a and S9b, we show the average Ti-$L_{2,3}$ edge XAS and XLD data (black line), normalized to the L_3-edge XAS intensity, and corresponding calculated spectra reproducing the data (red line). We also show in Figure S9b the calculated XLD expected in the case of simple tetragonal distortion (see table S1).

The data and the comparison with the calculated spectra show clearly that the Δt_{2g} and Δe_g splitting is opposite in both cases, with Ti-3d_{xy} and Nb-4d_{xy} lower in energy than Ti-3$d_{xz,yz}$ and Nb-4$d_{xz,yz}$, respectively, while Ti-3d_{x2-y2} and Nb-4d_{x2-y2} are higher in energy respect Ti-3d_{z2} and Nb-4d_{z2} states. This orbital hierarchy proves that NbO_6 and TiO_6 clusters are non-centrosymmetric, a signature of ferroelectric-like distortions in both KNN and interfacial STO unit-cells where the 2DEG resides.

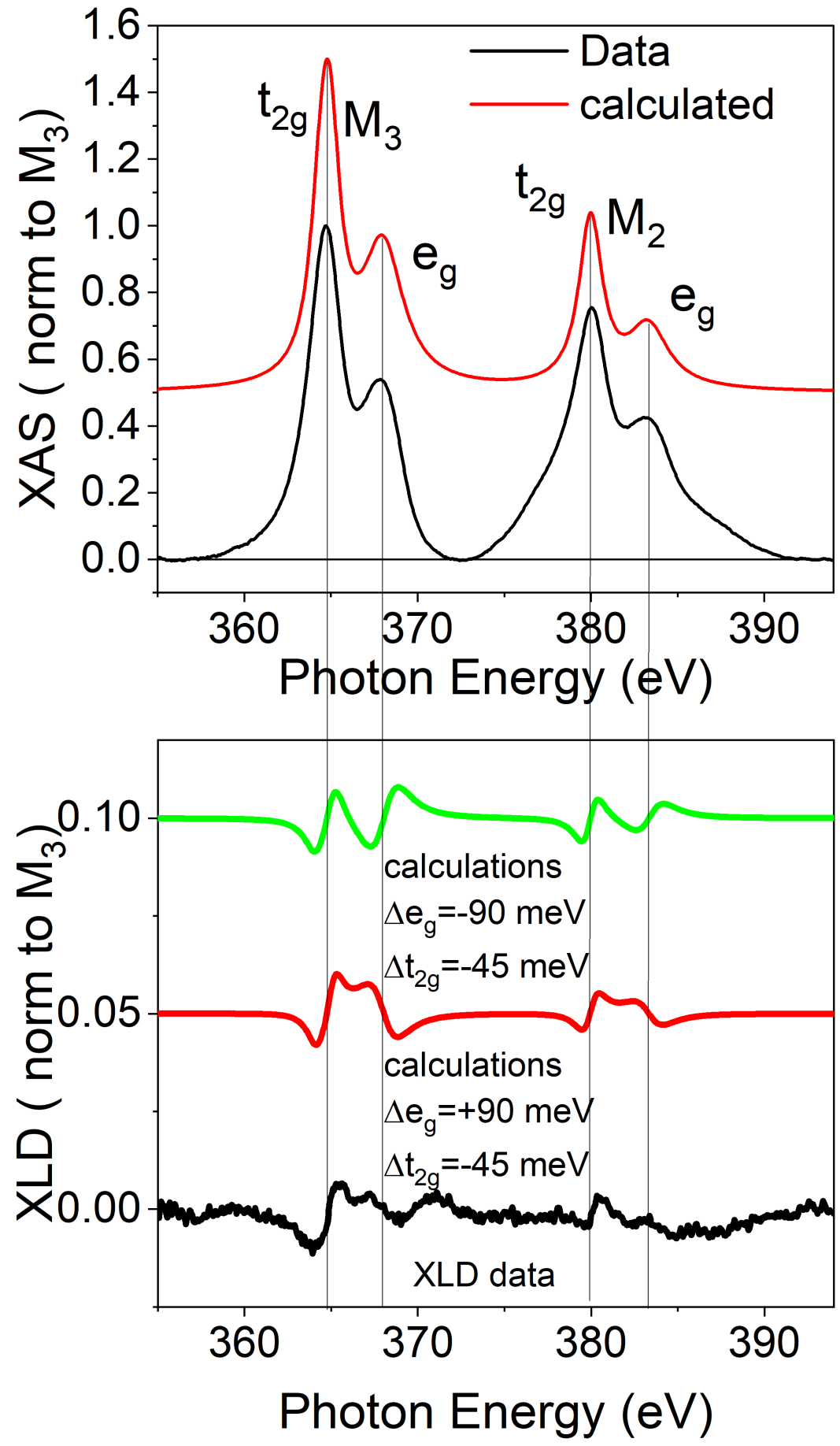

Figure S8: XAS, XLD atomic multiplet scattering simulation of Nb $M_{2,3}$-edge spectra. Upper panel: XAS data (black line) and calculated (red line) spectra normalized to M_3. The calculated XAS spectra have been shifted for clarity. Lower panel: XLD data (black line), calculated (red line) XLD spectrum reproducing the data, and calculated XLD spectrum assuming a simple tetragonal (centro-symmetric) distortion (green line) normalized to M_3. The calculated XLD spectra have been shifted for clarity.

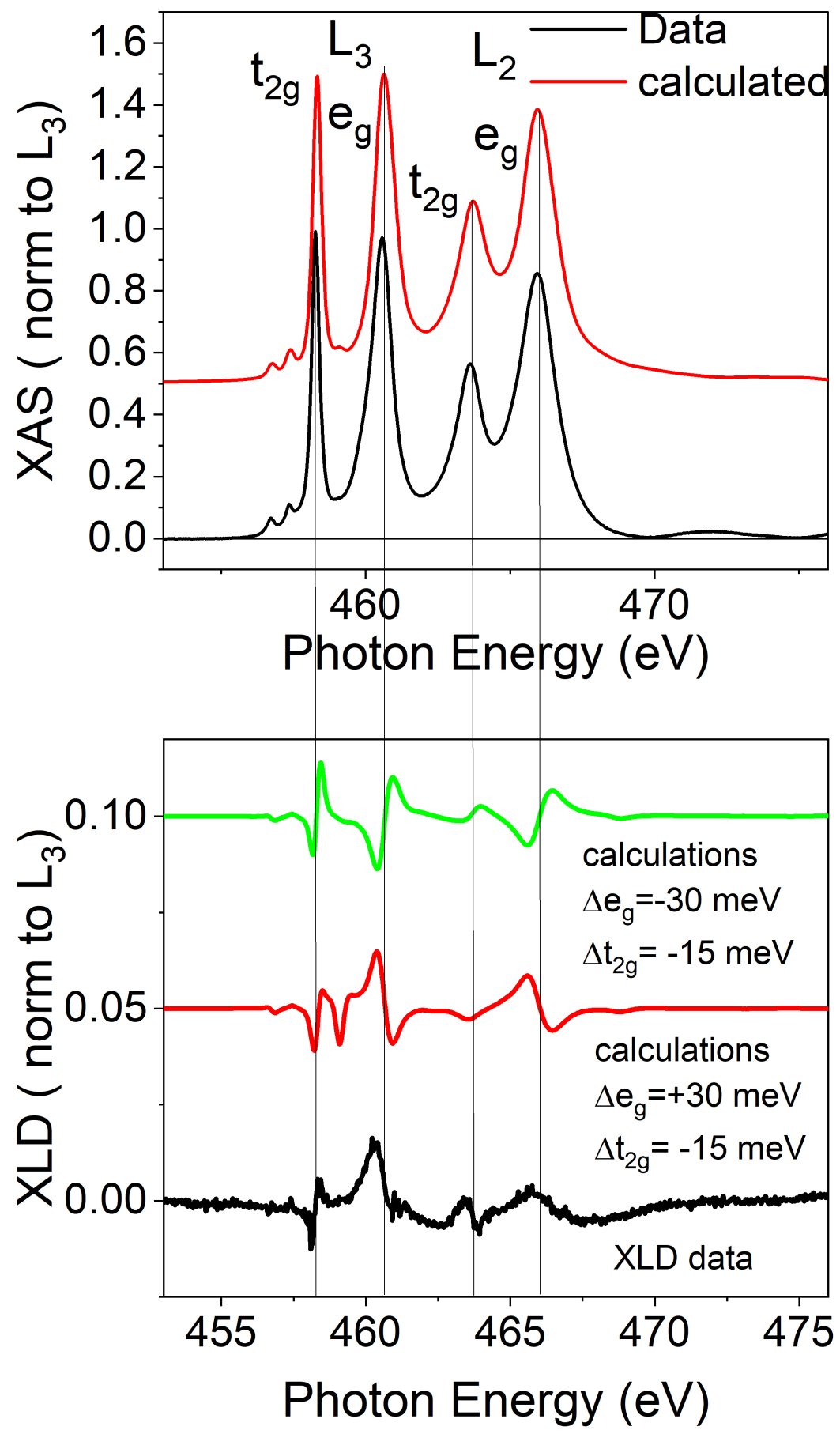

Figure S9: XAS, XLD atomic multiplet scattering simulation of Ti $L_{2,3}$-edge spectra. Upper panel: XAS data (black line) and calculated (red line) spectra normalized to L_3. The calculated XAS spectra have been shifted for clarity. Lower panel XLD data (black line), calculated (red line) XLD spectrum reproducing the data, and calculated XLD spectrum assuming a simple tetragonal (centro-symmetric) distortion (green line) normalized to L_3. The calculated XLD spectra have been shifted for clarity.

Note S6: Description of Second Harmonic Generation (SHG)

SHG is a nonlinear optical process in which two photons of identical frequency ω interact with a material, resulting in the emission of a single photon with doubled frequency 2ω. This phenomenon can be described by the following equation:

$$P_i(2\omega) = \varepsilon_0 \chi^{(2)}_{ijk} E^j(\omega) E^k(\omega)$$

Here, $P_i(2\omega)$ represents the nonlinear optical polarization, $\chi^{(2)}_{ijk}$ is the second-order nonlinear optical susceptibility, $E^{j,k}(\omega)$ are the components of the incident electric field within the material, and ε_0 is the vacuum permittivity.

SHG is highly sensitive to crystal symmetry: centrosymmetric crystals under dipole approximation exhibit no SHG, whereas crystals lacking inversion symmetry can generate photons at twice the input frequency. It is worth noting that also interfaces between symmetric crystals break the inversion symmetry leading to SHG generation. Importantly, phase transitions can break inversion symmetry, making SHG a powerful probe for studying such transitions. Ferroelectrics are a prototypical example where SHG is widely applied.

One of the crucial arguments to demonstrate the KNN layer is ferroelectric is the demonstration that the SHG signal increases proportionally to the KNN thickness.

We now discuss possible alternative mechanism and give decisive arguments to exclude alternative scenarios. Oxygen vacancies, in particular, is a possible source of SHG signal in some specific cases. The mere presence of oxygen vacancies is not, by itself, sufficient to generate an SHG signal. Oxygen vacancies acting as electron donors, can generate free electrons that can accumulate at the interface, giving rise to an interfacial electric field that might drive SHG emission through effective field coupling [6].

On this basis, we can identify three possible scenarios and test whether any of them is consistent with our observations: (1) KNN is ferroelectric and the oxygen-vacancy density is negligible; (2) KNN is ferroelectric, but the oxygen-vacancy density is non-negligible; and (3) KNN is not ferroelectric, and the observed SHG arises solely from oxygen vacancies.

The first scenario is the one we proposed, and it accounts for the experimental data as discussed in the manuscript.

In the second scenario, the free electrons introduced by oxygen vacancies would be expected to screen the ferroelectric polarization of KNN, leading to a negative slope in the SHG dependence on KNN thickness. This is exactly the opposite of what we observe experimentally, and this scenario can therefore be ruled out without hesitation.

The third scenario is also inconsistent with the data. Such a high free-charge density should produce a carrier concentration that scales with KNN thickness, and the sheet resistance should show a corresponding thickness dependence. However, neither effect is observed, as shown in Table S2 below. Considering also the growth conditions, which are not favourable to the formation of oxygen vacancies, and all other evidence given in the manuscript about ferroelectricity of the KNN, we conclude that the experimental evidence observed here cannot be attributed to oxygen vacancies.

KNN unit cells	R_{square} (kΩ)	n_{2D} (cm^{-2}) x10^{13}	μ ($cm^2V^{-1}s^{-1}$)
4	51	3.3	3.6
6	54	3.4	3.4
10	64	2.1	4.7

Table S2: KNN thickness dependence of the transport properties at room temperature

Note S7: Macroscopic ferroelectric measurements

We performed polarization–electric field measurements using a TF Analyzer (aixACCT).

To study the ferroelectric polarization of the KNN film, we fabricated capacitor structures by depositing 50 × 50 µm² Cr/Au top-electrodes on aLAO/KNN(6uc)/STO_{film}/STO_{SC}, while the 2DEG was used as bottom electrode.

To reduce/eliminate the influence of leakage current in the low thickness KNN dielectric/ferroelectric capacitor structure, we have used the active leakage compensation feature of our TF Analyzer system.

As shown in Fig. S10 below, despite the challenges associated with the ultrathin KNN layer and non-negligible leakage, the measurements reveal clear switching current peaks in the I–V characteristics, a well-defined polarization hysteresis and a saturation polarization of ~8 µC/cm². This provides direct and independent macroscopic evidence of ferroelectric switching**.**

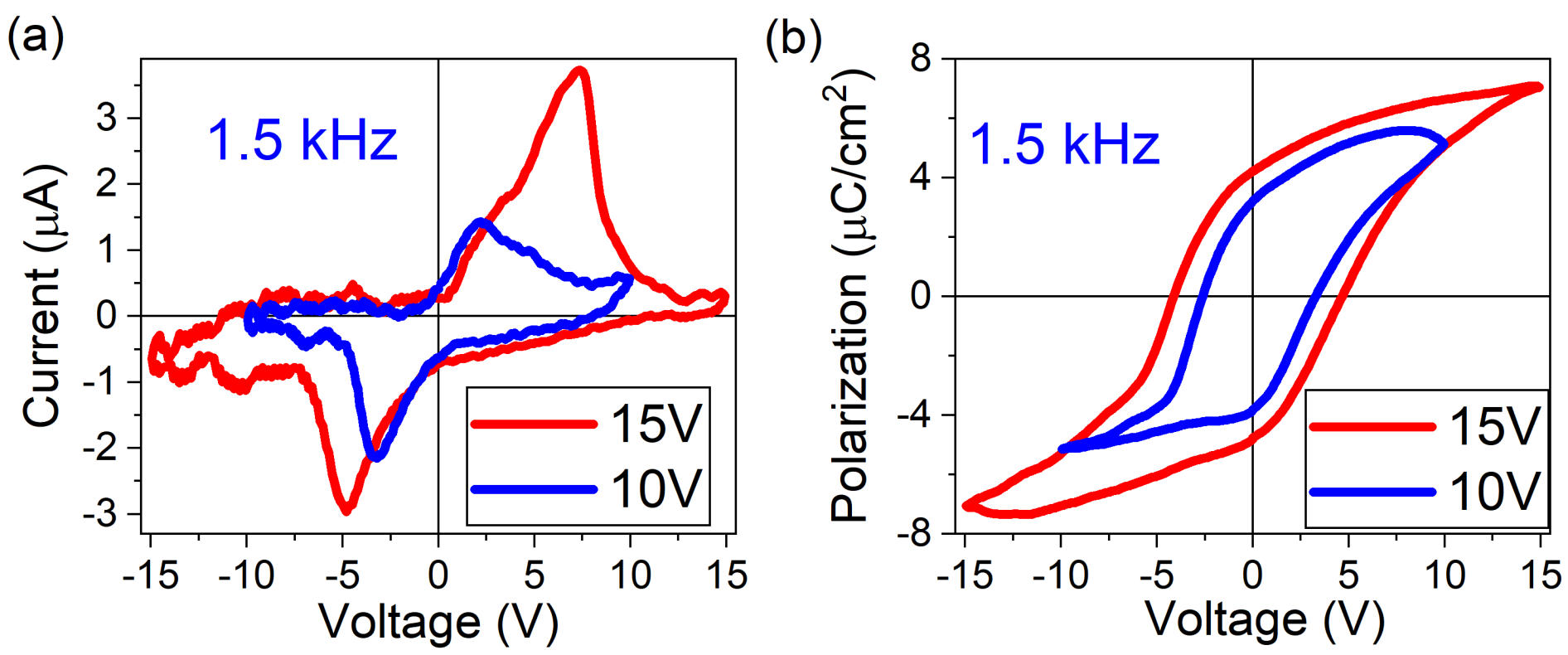

Figure S10: Macroscopic ferroelectric characterization of KNN films: (a) Current (I) and (b) Polarization vs. V_{gate} hysteresis loops measured between top-gate electrodes and the 2DEG of a-LAO (2 nm)/KNN (2.5 nm)/STO_{film} (10uc)/STO_{SC} film, at room temperature.

According to the experimental STEM and XLD data, FE-dipoles are observed also in the STO_{film} interfacial layer where the 2DEG is located. In order to determine if these dipoles are switchable, we have performed low temperature FE-polarization measurements using back gating configuration. Here the gate voltage was applied to the back-gate deposited on the STO substrate while the 2DEG was used as second electrode.

In this configuration, the KNN FE-layer is not involved in the switching process.

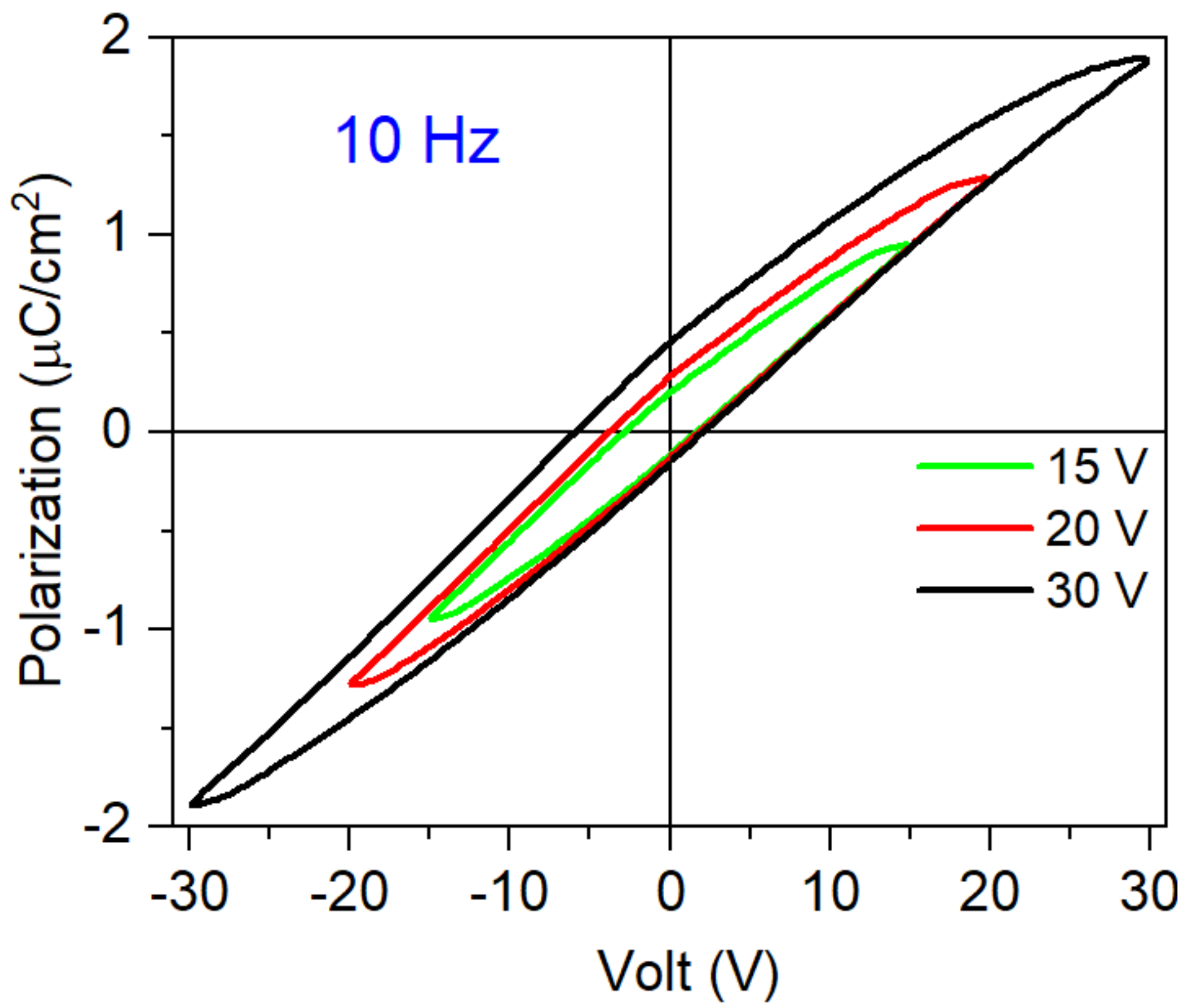

Figure S11: Macroscopic ferroelectric characterization in back gate configuration: Polarization vs. V_{gate} hysteresis loops measured between bottom-gate electrode and the 2DEG of a-LAO (2 nm)/KNN (2.5 nm)/STO_{film} (10uc)/STO_{SC} film, at 5K with different voltage ranges.

In Fig. S11 we show the FE-Polarization vs gate voltage loop measured at 10Hz using the TF analyzer. No leakage compensation was needed in this case, as the leakage was negligible due to the very large thickness of the substrate. The data show that there is a switchable polarization in the system.

Supplementary References

[1] Kobayashi, D. *et al.*, Sr surface segregation and water cleaning for atomically controlled $SrTiO_3$ (001) substrates studied by photoemission spectroscopy., *J. Electron Spectrosc. Relat. Phenom.,* **144-147**, 443–446, (2005).

[2] Bachelet, R., Sànchez, F., Palomares, F. J., Ocal, C. & Fontcuberta, J. Atomically flat SrO-terminated $SrTiO_3$(001) substrate., *Appl. Phys. Lett. ,* **95**, 141915, (2009).

[3] Baeumer, C., Xu, C., Gunkel, F. *et al.*, Surface Termination Conversion during $SrTiO_3$ Thin Film Growth Revealed by X-ray Photoelectron Spectroscopy," *Sci Rep.,* **5**, 11829, (2015).

[4] Cancellieri, C., Reinle-Schmitt, M. L., Kobayashi, M. *et.al.* Interface Fermi States of $LaAlO_3/SrTiO_3$ and Related Heterostructures, *Phys. Rev. Lett.,* **110**, 13, 137601, (2013).

[5] Stavitski, E. & de Groot, F. M., The CTM4XAS program for EELS and XAS spectral shape analysis of transition metal L edges., *Micron,* **41**, 687–694, (2010).

[6] Rubano A. & Paparo D., Optical Second Harmonic Generation on $LaAlO_3/SrTiO_3$ Interfaces: A Review., *Materials ,* **16**, 12, 4337, (2023).
